# Information from Pharmaceutical Companies and the Quality, Quantity, and Cost of Physicians' Prescribing: A Systematic Review

**DOI:** 10.1371/journal.pmed.1000352

**Published:** 2010-10-19

**Authors:** Geoffrey K. Spurling, Peter R. Mansfield, Brett D. Montgomery, Joel Lexchin, Jenny Doust, Noordin Othman, Agnes I. Vitry

**Affiliations:** 1 University of Queensland, Brisbane, Queensland, Australia; 2 Healthy Skepticism, Willunga, South Australia; 3 University of Adelaide, Adelaide, South Australia, Australia; 4 School of Primary, Aboriginal and Rural Health Care, University of Western Australia, Perth, Western Australia, Australia; 5 School of Health Policy and Management, York University, Toronto, Ontario, Canada; 6 Faculty of Health Sciences and Medicine, Bond University, Gold Coast, Queensland, Australia; 7 Kulliyyah of Pharmacy, International Islamic University Malaysia, Kuantan, Pahang, Malaysia; 8 Quality Use of Medicines and Pharmacy Research Centre, Sansom Institute, School of Pharmacy and Medical Sciences, University of South Australia, Adelaide, South Australia, Australia; Institute for Clinical Evaluative Sciences, Canada

## Abstract

**Background:**

Pharmaceutical companies spent $57.5 billion on pharmaceutical promotion in the United States in 2004. The industry claims that promotion provides scientific and educational information to physicians. While some evidence indicates that promotion may adversely influence prescribing, physicians hold a wide range of views about pharmaceutical promotion. The objective of this review is to examine the relationship between exposure to information from pharmaceutical companies and the quality, quantity, and cost of physicians' prescribing.

**Methods and Findings:**

We searched for studies of physicians with prescribing rights who were exposed to information from pharmaceutical companies (promotional or otherwise). Exposures included pharmaceutical sales representative visits, journal advertisements, attendance at pharmaceutical sponsored meetings, mailed information, prescribing software, and participation in sponsored clinical trials. The outcomes measured were quality, quantity, and cost of physicians' prescribing. We searched Medline (1966 to February 2008), International Pharmaceutical Abstracts (1970 to February 2008), Embase (1997 to February 2008), Current Contents (2001 to 2008), and Central (The Cochrane Library Issue 3, 2007) using the search terms developed with an expert librarian. Additionally, we reviewed reference lists and contacted experts and pharmaceutical companies for information. Randomized and observational studies evaluating information from pharmaceutical companies and measures of physicians' prescribing were independently appraised for methodological quality by two authors. Studies were excluded where insufficient study information precluded appraisal. The full text of 255 articles was retrieved from electronic databases (7,185 studies) and other sources (138 studies). Articles were then excluded because they did not fulfil inclusion criteria (179) or quality appraisal criteria (18), leaving 58 included studies with 87 distinct analyses. Data were extracted independently by two authors and a narrative synthesis performed following the MOOSE guidelines. Of the set of studies examining prescribing quality outcomes, five found associations between exposure to pharmaceutical company information and lower quality prescribing, four did not detect an association, and one found associations with lower and higher quality prescribing. 38 included studies found associations between exposure and higher frequency of prescribing and 13 did not detect an association. Five included studies found evidence for association with higher costs, four found no association, and one found an association with lower costs. The narrative synthesis finding of variable results was supported by a meta-analysis of studies of prescribing frequency that found significant heterogeneity. The observational nature of most included studies is the main limitation of this review.

**Conclusions:**

With rare exceptions, studies of exposure to information provided directly by pharmaceutical companies have found associations with higher prescribing frequency, higher costs, or lower prescribing quality or have not found significant associations. We did not find evidence of net improvements in prescribing, but the available literature does not exclude the possibility that prescribing may sometimes be improved. Still, we recommend that practitioners follow the precautionary principle and thus avoid exposure to information from pharmaceutical companies.

*Please see later in the article for the Editors' Summary*

## Abstract 2

Geoff Spurling and colleagues report findings of a systematic review looking at the relationship between exposure to promotional material from pharmaceutical companies and the quality, quantity, and cost of prescribing. They fail to find evidence of improvements in prescribing after exposure, and find some evidence of an association with higher prescribing frequency, higher costs, or lower prescribing quality.

## Editors' Summary

**Background:**

A prescription drug is a medication that can be supplied only with a written instruction (“prescription”) from a physician or other licensed healthcare professional. In 2009, 3.9 billion drug prescriptions were dispensed in the US alone and US pharmaceutical companies made US$300 billion in sales revenue. Every year, a large proportion of this revenue is spent on drug promotion. In 2004, for example, a quarter of US drug revenue was spent on pharmaceutical promotion. The pharmaceutical industry claims that drug promotion—visits from pharmaceutical sales representatives, advertisements in journals and prescribing software, sponsorship of meetings, mailed information—helps to inform and educate healthcare professionals about the risks and benefits of their products and thereby ensures that patients receive the best possible care. Physicians, however, hold a wide range of views about pharmaceutical promotion. Some see it as a useful and convenient source of information. Others deny that they are influenced by pharmaceutical company promotion but claim that it influences other physicians. Meanwhile, several professional organizations have called for tighter control of promotional activities because of fears that pharmaceutical promotion might encourage physicians to prescribe inappropriate or needlessly expensive drugs.

**Why Was This Study Done?:**

But is there any evidence that pharmaceutical promotion adversely influences prescribing? Reviews of the research literature undertaken in 2000 and 2005 provide some evidence that drug promotion influences prescribing behavior. However, these reviews only partly assessed the relationship between information from pharmaceutical companies and prescribing costs and quality and are now out of date. In this study, therefore, the researchers undertake a systematic review (a study that uses predefined criteria to identify all the research on a given topic) to reexamine the relationship between exposure to information from pharmaceutical companies and the quality, quantity, and cost of physicians' prescribing.

**What Did the Researchers Do and Find?:**

The researchers searched the literature for studies of licensed physicians who were exposed to promotional and other information from pharmaceutical companies. They identified 58 studies that included a measure of exposure to any type of information directly provided by pharmaceutical companies and a measure of physicians' prescribing behavior. They then undertook a “narrative synthesis,” a descriptive analysis of the data in these studies. Ten of the studies, they report, examined the relationship between exposure to pharmaceutical company information and prescribing quality (as judged, for example, by physician drug choices in response to clinical vignettes). All but one of these studies suggested that exposure to drug company information was associated with lower prescribing quality or no association was detected. In the 51 studies that examined the relationship between exposure to drug company information and prescribing frequency, exposure to information was associated with more frequent prescribing or no association was detected. Thus, for example, 17 out of 29 studies of the effect of pharmaceutical sales representatives' visits found an association between visits and increased prescribing; none found an association with less frequent prescribing. Finally, eight studies examined the relationship between exposure to pharmaceutical company information and prescribing costs. With one exception, these studies indicated that exposure to information was associated with a higher cost of prescribing or no association was detected. So, for example, one study found that physicians with low prescribing costs were more likely to have rarely or never read promotional mail or journal advertisements from pharmaceutical companies than physicians with high prescribing costs.

**What Do These Findings Mean?:**

With rare exceptions, these findings suggest that exposure to pharmaceutical company information is associated with either no effect on physicians' prescribing behavior or with adverse affects (reduced quality, increased frequency, or increased costs). Because most of the studies included in the review were observational studies—the physicians in the studies were not randomly selected to receive or not receive drug company information—it is not possible to conclude that exposure to information actually causes any changes in physician behavior. Furthermore, although these findings provide no evidence for any net improvement in prescribing after exposure to pharmaceutical company information, the researchers note that it would be wrong to conclude that improvements do not sometimes happen. The findings support the case for reforms to reduce negative influence to prescribing from pharmaceutical promotion.

**Additional Information:**

Please access these Web sites via the online version of this summary at http://dx.doi.org/10.1371/journal.pmed.1000352.

- Wikipedia has pages on prescription drugs and on pharmaceutical marketing (note that Wikipedia is a free online encyclopedia that anyone can edit; available in several languages)
- The UK General Medical Council provides guidelines on good practice in prescribing medicines
- The US Food and Drug Administration provides information on prescription drugs and on its Bad Ad Program
- Healthy Skepticism is an international nonprofit membership association that aims to improve health by reducing harm from misleading health information
- The Drug Promotion Database was developed by the World Health Organization Department of Essential Drugs & Medicines Policy and Health Action International Europe to address unethical and inappropriate drug promotion

## Introduction

Pharmaceutical companies in the United States spent about US$57.5 billion, or 24.4% of their revenue, on promotion in 2004 [1]. One estimate of total promotional expenditure in France for 2004 is €2,908 million (12.2% of revenue). However, another estimate is that pharmaceutical detailing cost €3,300 million and accounted for 75% of the overall cost of promotion in that year making promotion 17.3% of revenue [2]. Expenditure on promotion is aimed at maximizing returns for the corporation and shareholders [3]. The industry claims that promotion also provides scientific and educational information to healthcare professionals: “Appropriate marketing of medicines ensures that patients have access to the products they need and that the products are used correctly for maximum patient benefit. Our relationships with healthcare professionals are critical to achieving these goals because they enable us to – inform healthcare professionals about the benefits and risks of our products to help advance appropriate patient use, provide scientific and educational information, support medical research and education” [4].

There is a wide range of views amongst health professionals about pharmaceutical promotion. Qualitative studies suggest that many perceive pharmaceutical promotion to be a useful and convenient source of information [5]–[7]. Some doctors deny that they are influenced by pharmaceutical company promotion or claim that it influences others but not themselves [8]–[10]. Nonetheless, many of these physicians are willing to give significant amounts of time to engaging in promotional activities [11]. By contrast, several professional organisations have called for more control of promotional activities [12],[13] because of evidence that promotion may be misleading [14]–[17].

The evidence base illuminating these conflicting views is growing. In 2000, Wazana identified eight studies linking pharmaceutical promotion to increased prescribing, “nonrational prescribing,” and increased prescribing costs [18]. A 2005 review concluded that promotion influences the prescribing by physicians in training [19], and a second review in the same year concluded that sales representatives influence prescribing [20].

Those previous reviews are now out of date, narrowly focused, or only partially assessed the relationship between information (promotional or otherwise) from pharmaceutical companies and prescribing costs and quality. The objective of this review is to examine the relationship between exposure to information directly provided by pharmaceutical companies and the quality, quantity, and cost of physicians' prescribing.

## Methods

### Criteria for Including Studies

Randomized controlled trials, time series analyses, before–after studies, cohort studies, case-control studies, ecological studies, and cross-sectional studies were eligible for inclusion. Studies were included if they had both a measure of exposure to any type of information directly provided by pharmaceutical companies and a measure of physicians' prescribing. We excluded studies that looked at the indirect provision of information, for example, through continuing medical education courses that were funded by unrestricted grants from pharmaceutical companies. Case series, case reports, abstracts, news items, and short reports were excluded.

Exposure to information directly provided by pharmaceutical companies was defined as including pharmaceutical sales representative visits, advertisements in journals or prescribing software, presentations from pharmaceutical companies to groups, meetings sponsored by pharmaceutical companies, mailed information including advertisements, and participation in sponsored clinical trials. We did not include studies of other forms of promotion such as gifts or samples or studies of indirect forms of information provision such as sponsored education.

The outcome measures were the quality, frequency, and costs of prescribing.

### Search Methods for Identification of Studies

We searched Medline (1966 to February 2008), International Pharmaceutical Abstracts (1970 to February 2008), Embase (1997 to February 2008), Current Contents (2001 to 2008), and Central (The Cochrane Library Issue 3, 2007). The search strategy below was devised for Medline by an expert librarian at the University of Queensland and adapted for the other databases: (exp Drug Industry OR exp Advertising OR exp Gift Giving OR exp “Conflict of Interest”) AND (exp Prescriptions, Drug/OR (prescribing or prescription$).mp.))

We looked for additional articles in the references of each retrieved article including review articles in an iterative, exhaustive process. Efforts to find additional studies included placement of messages on email drug discussion groups, contacting experts in the field, and asking Australian subsidiaries of international pharmaceutical companies for information. All languages were considered.

### Selection of Studies

The title and abstract, if available, of all articles detected by the database searches were reviewed by two authors. Articles that possibly met the inclusion criteria were retrieved and subjected to a formal inclusion process independently by two different authors. Differences of opinion were resolved by consensus and if necessary a third author was involved.

### Quality Appraisal

Articles meeting inclusion criteria were appraised for methodological quality independently by two authors. Randomized studies were assessed for adequacy of randomization method, allocation concealment, blinding, follow-up, and use of intention to treat analyses [21]. Controlled cohort and case-control studies were assessed using the Newcastle-Ottawa scales [22]. For other nonrandomized studies, quality appraisal included assessment of sources of bias, for example presence of a control group, selection methods, control of confounding, response rate (>80%), and use of appropriate statistical tests [23]. Studies were only excluded from the review if two authors found there was insufficient information to appraise their quality. Disagreements were resolved by discussion with a third author.

### Data Extraction

For included studies, two authors independently extracted data on study site, dates of data collection or publication, types of participants (primary care providers, specialists, and residents), study medication(s), exposure to information from pharmaceutical companies, and prescribing outcomes.

### Reporting of Results

For quality of prescribing we accepted the original authors' definitions of what constituted more (or less) appropriate prescribing.

We divided studies into two groups on the basis of whether the information was delivered with or without conventional promotional techniques. This distinction was made because information delivered with versus without conventional promotion may produce different effects on prescribing.

Conventional promotional techniques were defined as advertisements (in journals and software), representatives' visits, attendance at pharmaceutical sponsored meetings, and mailed information from pharmaceutical companies. In addition, we included in this group studies looking at total promotional investment/summated scores of commercial information use/general use of commercial sources. The other group of studies included warning letters, participation in company sponsored trials, and representatives' visits for nonpromotional purposes.

A narrative synthesis of results was undertaken following the MOOSE guidelines and meta-analysis performed where appropriate data were available (Text S1) [24]. The unit of analysis was defined as the combination of exposure to a type of information from a pharmaceutical company (for example pharmaceutical sales representative visits or journal advertisements) and a type of prescribing outcome (quality, frequency, and cost of prescribing). Thus studies were treated as a single unit of analysis if they measured the same type of exposure and the same type of outcome regardless of the number of drugs covered in each study. We classified each analysis as positive or negative rather than no association detected if the *p* value was less than 5% (*p*<0.05) regardless of the magnitude of the effect.

We reported standardized effect measures (Pearson correlation coefficients, odds ratios [ORs], or beta coefficients) where study reports provided them or the data needed to calculate them. For econometric studies, we also reported *t* statistics where they were reported or it was possible to calculate them.

Meta-analysis was not appropriate for the outcomes of quality of prescribing and cost of prescribing because in both cases the studies examined different exposures or outcome measures and/or lacked control groups. We undertook a meta-analysis for one component—studies of frequency of prescribing with identifiable control groups where the information exposure was delivered with conventional promotional techniques. We used ORs for change in prescribing frequency as the outcome measure. Where studies had suitable designs for inclusion in the meta-analysis but ORs and standard errors were not published we contacted corresponding authors. Out of ten studies [25]–[34], we received four replies of which three provided the information we required [29]–[31].

Heterogeneity was assessed using the tau squared test with a sensitivity analysis to investigate likely sources of heterogeneity. Factors identified a priori as possible explanations for heterogeneity were study design, study quality indicators, year of publication, type of exposure to pharmaceutical company information (active versus passive), and physician characteristics (level of experience and also primary care provider versus specialist). We defined active exposure as information presented to physicians at meetings or during pharmaceutical sales representatives' visits. We defined passive exposure as journal advertisements, mailed information, advertisements on clinical software, and participation in sponsored clinical trials. Studies reporting more than one unit of analysis were subjected to sensitivity analysis. Meta-analysis was performed using RevMan (version 5.0.24) with further analysis conducted using Stata version 10.0 (Stata Corporation).

## Results

### Search Results

**Figure 1:**
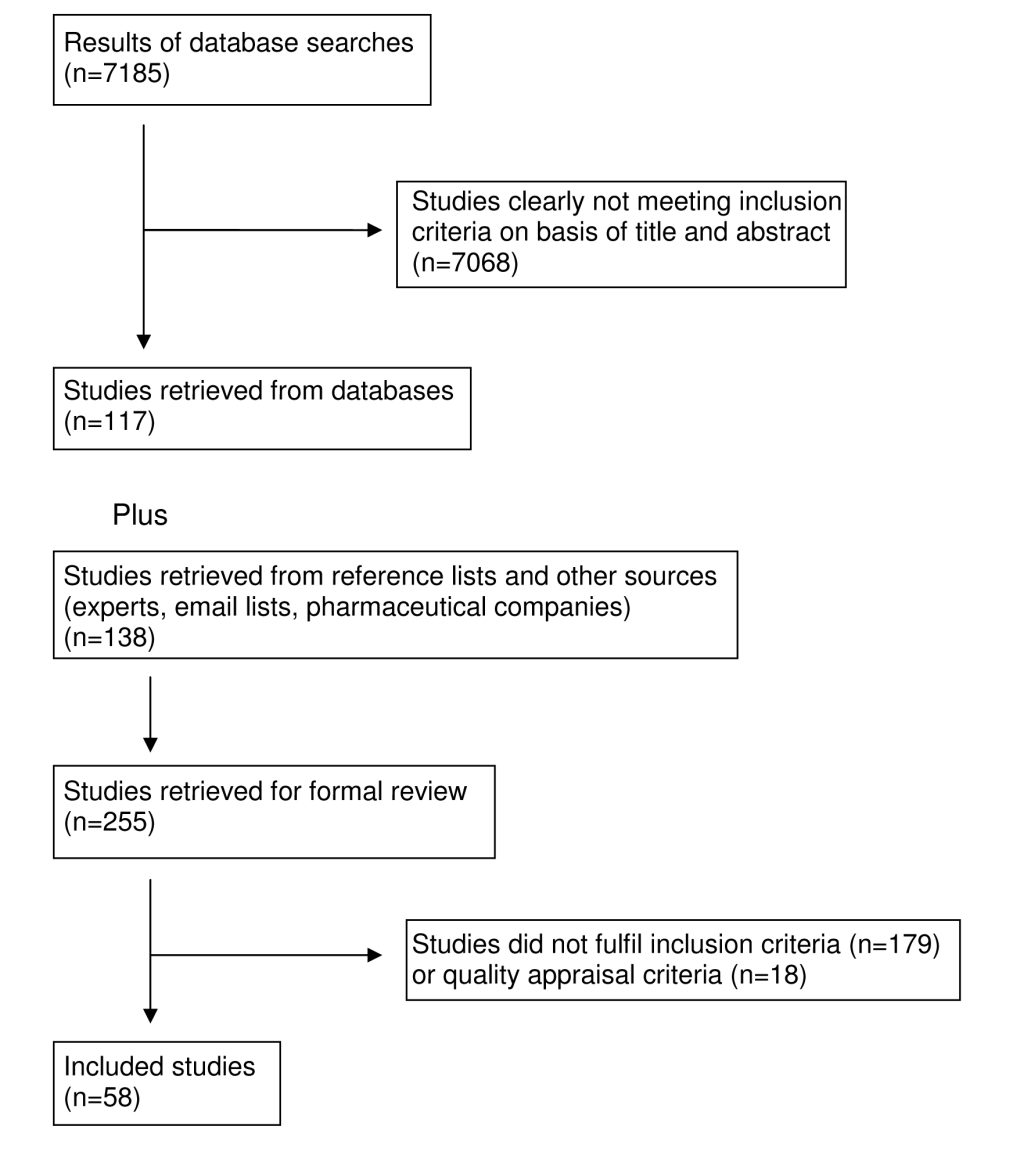
Study flow diagram.

Our search found 7,185 studies from electronic databases and 138 studies were retrieved from reference lists, experts in the field and email lists. The full text of 255 articles was retrieved. 18 studies were excluded, all because inadequate reporting precluded quality assessment. Quality appraisal results for included studies are presented in Tables 1–5. Following application of inclusion/exclusion criteria and quality appraisal, 58 studies were included in the review (52 published in journals [25]–[33],[35]–[77], three reports [78]–[80], one dissertation [34], one conference presentation [81], and one conference poster [82] (Figure 1). Of these 58, 29 studies came from database searches [25]–[31],[33],[35]–[38],[41],[44],[55],[56],[59]–[62],[66]–[68],[70]–[72],[74],[76],[77], 22 studies came from reference lists [32],[39],[40],[46]–[54],[58],[63]–[65],[69],[73],[75],[78],[79], five studies came from experts in the field [34],[43],[57],[81],[82], and two from email lists [45],[80]. These 58 studies included 87 units of analysis. Pharmaceutical companies provided 62 citations; two of these met our inclusion criteria and had already been identified through Medline searches [27],[35]. Five of the studies located through the e-mail lists and experts were not indexed in the databases we searched [34],[43],[80]–[82]. For one study [78], additional data were obtained from the authors [83].

**Table 1:** Quality appraisal of included studies: randomised controlled trials.

Randomised Controlled Study (First Author Name)	Satisfactory Randomization	Allocation Concealment	Blinding	Adequate Follow-up	Appropriate Statistical Measures
**Freemantle ** [**35**]	Appropriate cluster randomization	No	No	Yes	Yes
**Dolovich ** [**36**] a	Appropriate cluster randomization	No	No	Yes	Yes

a Received research funding from a pharmaceutical company.

**Table 2 pmed-1000352-t002:** Quality appraisal of included studies: controlled cohort and case-control studies.

Study Type	Study (First Author Name)	Prospective Design	Comparability of Cases and Controls	Selection Bias Minimized	Response Rate >80%	Confounders Controlled	Appropriate Statistical Measures	Adequate Follow-Up
Controlled Cohort	Andersen [37] a	No	Yes	Yes	Yes	Yes	Yes	Yes
Case-Control	Spingarn [39]	No	Yes	No	Yes (100%)	Yes	Yes	Yes
	Chren [38]	No	Yes	Yes	Yes (88%)	Yes	Yes	Yes

a Received research funding from a pharmaceutical company.

**Table 3 pmed-1000352-t003:** Quality appraisal of included studies: time-series analyses.

Time-Series Analysis	Study (First Author Name)	Prospective Design	Control Group	Confounders Controlled	Selection Bias Minimized	Appropriate Statistical Measures
**Econometric**	Ching [78]	No	No	Yes	Yes	Yes
	Venkataraman [40]	No	No	Yes	Yes	Yes
	Windmeijer [41]	No	No	Yes	Yes	Yes
	Chintagunta [42]	No	No	Yes	Yes	Yes
	Narayanan [43]	No	No	Yes	Yes	Yes
	Donohue [44]	No	No	Yes	Yes	Yes
	Mizik [45]	No	No	Yes	Yes	Yes
	Manchanda [46]	No	No	Yes	Yes	Yes
	Manchanda and Chintagunta [47]	No	No	Yes	Yes	Yes
	Berndt [48]	No	No	Yes	Yes	Yes
	Rosenthal [79]	No	No	Yes	No	Yes
	Azoulay [49]	No	No	Yes	Yes	Yes
	Rizzo [50]	No	No	Yes	No	Yes
	Hurwitz [51]	No	No	Yes	Yes	Yes
	Mackowiak [52]	No	No	No	Yes	Yes
	Leffler [53]	No	No	Yes	Yes	Yes
	Telser [54]	No	No	Yes	Yes	Yes
**Other**	Spurling [55]	Yes	No	No	No	Yes
	Stafford [56]	Yes	No	No	Yes	No
	Charbit [34]	No	No	No	Yes	No
	Auvray [57]	No	No	No	No	No
	Cleary [26]	Yes	Yes	Yes	No	Yes
	Soumerai [58]	No	Yes	No	Yes	No

**Table 4 pmed-1000352-t004:** Quality appraisal of included studies: before–after studies.

Before–After Study (First Author Name)	Prospective Design	Control Group	Response Rate >80%	Confounders Controlled	Selection Bias Minimized
Hemminki [25]	No	Yes	No (68%)	No	Yes
Schwartz [27]	No	Yes	Unsure	No	Unsure
Kazmierczak [59]	No	No	NA	No	Yes
Orlowski [28]	No	No	Yes (100%)	Yes	No
Bowman [60]	Yes	No	No (43%–77%)	No	No

**Table 5:** Quality appraisal of included studies: cross-sectional studies (no control group).

Cross-Sectional Study (First Author Name)	Prospective Design	Response Rate >80%	Confounders Controlled	Selection Bias Minimized	Appropriate Statistical Measures
Henderson [29] a	No	Yes	Yes	Yes	Yes
Greving [30]	No	Yes (96%)	Yes	Yes	Yes
Kreyenbuhl [31]	Yes	No (58%)	No	Yes	Yes
de Bakker [61]	No	Unsure	Yes	Yes	Yes
Steinman [62]	No	Yes	Yes	No	Yes
Canli [32]	Yes	No (79%)	No	Yes	Yes
Verdoux [63]	Yes	No (24%)	Yes	No	Yes
Muijrers [64]	Yes	No (71%)	Yes	Yes	Yes
Huang [65]	No	NA	No	No	Yes
Watkins [66]	Yes	No (64%)	Yes	Yes	Yes
Prosser [67]	Yes	No (73%)	No	Yes	No
Caamano [68]	Yes	No (75%)	Yes	Yes	Yes
Gonul [69]	Yes	NA	Yes	Unsure	Yes
Mansfield [82]	Yes	No (6%)	No	No	Yes
Jones [70]	Yes	NA	No	No	No
Caudill [71]	Yes	No (28%)	Yes	Yes	Yes
Berings [72]	Yes	No (28%)	Yes	No	Yes
Lurie [73]	Yes	No (75–78%)	Yes	Yes	Yes
Health Care Communications 1989^a^ [80]	No	Unsure	No	No	No
Peay [33]	No	No (52%–70%)	Yes	Yes	Yes
Blondeel [81]	Yes	No (30%)	Yes	Yes	Yes
Haayer [74]	Yes	Yes (90%)	Yes	No	Yes
Walton [75]	Yes	Unsure	No	Yes	Yes
Dajda [76]	No	NA	No	Yes	Yes
Becker [77]	Yes	Yes (84%)	Yes	Yes	Yes

a Received research funding from a pharmaceutical company.

### General Characteristics of Studies

The most common study design was cross-sectional (24/58 studies, 41%). There were also two cluster randomized controlled trials, one controlled-cohort study, two case-control studies, 24 time-series analyses, and five before–after studies. Over half (55%) of the studies were conducted in the United States. Characteristics of included studies are outlined in Table 6.

**Table 6:** Characteristics of included studies (by study design, year of publication, then sample size).

Study Design	Study (First Author Name)	Study Site, Year	Participants (*n*)	Medication	Intervention/Exposure	Outcome Measure(s)
**RCT**	Freemantle [35] a	UK 2000	PCPs (79: 40 intervention, 39 control)	Lansoprazole versus omeprazole	PSR visits: PSRs instructed by local health authority (one visit); controls: normal detailing	Switch from omeprazole to less costly lansoprazole
	Dolovich [36] a	Canada 1999	PCPs and pediatric specialists (641 in intervention group and 574 in control group)	Antibiotics for otitis media	PSR visits, PSRs trained in evidence-based education by academic department of a university; Control group: no detailing	Market share of antibiotics for otitis media
**Controlled cohort studies**	Andersen [37] b	Denmark 1999–2003	297 PCPs (26 intevention/271 controls)	Asthma medications	Participation in a RCT funded by a pharmaceutical company	Prescribing trial drug; Adherence to prescribing guidelines
**Case-control Studies**	Spingarn [39]	US° 1990	Hospital residents (75)	Medications for Lyme disease	Intervention: presentation by academic who was also a pharmaceutical executive; Controls: did not attend	Appropriateness of intention to prescribe for mild versus severe Lyme disease
	Chren [38]	US 1989–1990	Physicians (40 cases, 80 controls)	Addition to hospital formulary	PSR visits; cases added to formulary, controls did not	Addition of detailed drug to hospital formulary
**Time series analyses (econometric)**	Ching [78] c	Canada 1993–1999	Physician's prescribing antihypertensives in Canada	Antihypertensive medications (angiotensin converting enzyme inhibitors and diuretics)	PSR visits (*n* minutes)	Market share; Elasticity of demand
	Venkataraman [40] b	Not stated 2002–2003	Physicians (2,774)	Statins, coagulation drugs, erectile dysfunction drugs, gastrointestinal drugs, placebo	PSR visits (total number); attendance at pharmaceutical; sponsored meetings; (total number attended)	*n* prescriptions
	Windmeijer [41] c	Netherlands 1995–1999	PCPs and psychiatrists^d^	11 therapeutic markets (over 50% of the Dutch drug market)	PSR visits (expenditure); Journal advertisements (expenditure); Mail (expenditure)	*n* prescriptions; Cost of prescriptions
	Chintagunta [42] c	US, UK, Germany, France, Italy 1989–1999	Prescribers of antidepressant medications	Fluoxetine, sertraline, paroxetine	PSR visits (expenditure)	Market share (sales)
	Narayanan [43] e	US 1993–2001	All prescribers of antihistamines in US^d^	2nd generation antihistamines: loratidine cetirizine, fexofenadine	PSR visits (total expenditure)	New prescriptions per month
	Donohue [44] c	US 1997–2000	11,000 office and hospital physicians	First prescriptions of 6 antidepressants	Monthly spending on PSR detailing	New prescriptions
	Mizik [45] b	US 2004	Physicians (74,075)	3 unknown drugs	PSR visits	*n* new prescriptions for the three study drugs
	Manchanda [46] b	US 1999–2001	Physicians (1,000), 18.5% specialists (for study drug), 60.1% PCPs, 21.4% other specialists, controls (1,000)	Drug unknown	PSR visits	Numbers of prescriptions
	Manchanda and Chintagunta [47] b	US 1996–1998	Physicians (1,000), 11% specialists (for study drug), 59% PCPs, 30% other specialists	Drug unknown	PSR visits	*n* prescriptions; Prescriptions of specialists versus primary care physicians versus other specialists; Prescriptions by male and female physicians
	Berndt [48] c	US 1977–1993	All US physicians	H2 antagonist antiulcer drugs (cimetidine, ranitidine, famotidine, nizatidine)	PSR visits (min)	Sales volume (units of average daily dose) and market share; Elasticity of demand
	Rosenthal [79] c	US 1996–1999	Large sample of office and hospital physicians^d^	Medications prescribed in primary care	PSR visits (expenditure)	Sales of detailed medications per month
	Azoulay [49] c	US 1977–1993	All prescribing physicians	H2 antagonist antiulcer drugs (cimetidine, ranitidine, famotidine, nizatidine)	PSR visits; Journal advertisements	Market share for the four H2 antagonists (patient days of therapy)
	Gonul [69] c	US 1989–1994	Physicians^d^	One medication for a particular indication “relatively more common among the elderly”	PSR visits (min)	*n* prescriptions; Cost of Prescriptions
	Rizzo [50] c	US 1988–1993	All prescribers of antihypertensives in the US^d^	Antihypertensive medications	PSR visits (expenditure)	Sales of detailed medication; Price elasticity; Quadratic term for sales
	Hurwitz [51] c	US 1978–1983	Specialists and PCPs prescribing promoted drugs^d^	Brand and generic drugs	Total promotional investment in PSR visits, journal advertising, direct mail advertising	Market share held by original brand; Market share held by generic competitors (measured in *n* pills sold)
	Mackowiak [52] c	US 1977–1981	Office based physicians across the US^d^	Benzodiazepines for anxiety; Diuretics for hypertension	PSR visits (expenditure); Journal advertisements (expenditure)	Expenditure on prescriptions; Market size and market share
	Leffler [53] c	US 1968–1977	Not stated^d^	51 new products	Total promotional outlay (PSR visits, journal advertising)	Market share 2 y after market entry; Market share in 1977 for drugs introduced since 1968 expressed
	Telser [54] c	US 1963–1972	All prescribing physicians^d^	Prescription medications in: the hospital market and drugstore market	Promotional intensity: ratio of total promotional outlays/total sales (includes PSR visits, journal advertising, direct mail)	Proportion of sales for entrant drugs
**Time series analyses (other)**	Spurling [55]	Australia 2004–2005	PCPs (7)	Medications prescribed in primary care	PSR visits; Promotional items in PCP surgeries	Generic prescribing (% of total)
	Stafford [56] c	US 1996–2002	Physicians (3,500)	Alpha-blockers	PSR visits (expenditure)	Prescriptions
	Charbit [34]	France 1991–2001	Prescribing physicians in France^d^	6 classes of antihypertensive medications	Journal advertising (*n* pages)	Drug sales for each of the six classes of antihypertensive medications
	Auvray [57] e	France 1992–1998	PCPs, ear nose throat surgeons, chest physicians, psychiatrists-1,600	Macrolide antibiotics and psychoanaleptic antidepressants	Total promotional investment	*n* prescriptions
	Cleary [26]	US 1988	Physicians prescribing 3rd generation cephalosporins^d^	Ceftazidime, cefriaxone, cefotaxime	PSR visits	New prescriptions; *n* doses
	Soumerai [58] e	US 1974–1983	All propoxyphene prescribers in USA^d^	Propoxyphene	PSR visits (to warn about risks of propoxyphene)	Sales of propoxyphene; No-refill rates of prescriptions
**Before–after Studies**	Hemminki [25] e	Estonia 2000	Gynecologists and PCPs (342)	Hormone replacement therapy	Journal advertisements; Pharmaceutical company-sponsored medical education	Probability of detailed drug being prescribed
	Schwartz [27]	US 1999–2000	Psychiatry residents^d^	Psychiatric medications	PSR detailing (12 wk period when residents were detailed versus 12 wk with no detailing)	New prescriptions
	Kazmierczak [59]	US 1996	Physicians (60)	Tramadol	Drug company letter to physicians warning about tramadol seizure risk	Prescriptions for tramadol in high risk patients
	Orlowski [28]	US 1992	Hospital physicians (20)	Intravenous hospital medications called A (antibiotic) and B (cardiovascular drug)	Attendance at pharmaceutical sponsored meetings (all expenses paid trips to vacation site)	*n* prescriptions before and after the sponsored meetings
	Bowman [60]	US date not stated	Physicians (374)	Calcium channel blockers and beta-blockers	PSR sponsored continuing medical education course	Self-reported new prescriptions
**Cross-sectional studies**	Henderson [29]	Australia 2003–2005	PCPs (1,336)	7 advertised pharmaceutical products	Advertising on clinical software	*n* prescriptions
	Kreyenbuhl [31]	US 2003–2004	Psychiatrists^d^	Antipsychotic medication	PSR visits; Attendance at pharmaceutical sponsored meetings	Use of “switch” or “add” strategies in treatment of refractory schizophrenia
	de Bakker [61]	Netherlands 2001	PCPs (138)	Medications prescribed in primary care	PSR visits; Reliance on commercial sources of information	*n* prescriptions
	Steinman [62]	US 1995–1990	Physicians (97)	Gabapentin	PSR visits	Intention to prescribe gabapentin
	Greving [30]	Netherlands 2003	PCPs (70)	Angiotensin II receptor blockers	PSR visits; Journal advertisements; Attendance at pharmaceutical sponsored meetings	New prescriptions of this drug
	Canli [32]	Turkey 2001	PCPs (316)	Antibiotics for acute tonsillopharyngitis	PSR visits	Intention to prescribe antibiotics
	Verdoux [63]	France 2004	PCPs (848)	Antipsychotic medication	PSR visits	Initiation of antipsychotic medication in a 1-mo period
	Muijrers [64]	Netherlands 2000–2001	PCPs (1,434)	Medications prescribed in primary care	PSR visits	Quality of prescribing (determined by panel of experts)
	Huang [65]	US 2001–2003	Resident physicians^d^	Antidepressants	Sponsorship of resident conferences	Prescription of antidepressants from sponsoring companies
	Watkins [66]	UK 1995–1996	PCPs (1,714)	Medications prescribed in primary care	PSR visits (at least once per week); Journal advertisements; Reading written material from pharmaceutical companies	Cost of prescriptions
	Prosser [67]	UK 1999–2000	PCPs (107)	New medications prescribed in primary care	PSR visits; Journal advertisements/mailings (considered together)	New drug prescriptions (high/medium/low prescribers)
	Caamano [68] e	Spain 1993	Physicians (234)	All prescribing	PSR visits	*n* prescriptions ; Cost of prescriptions
	Mansfield [82]	Australia 1999	PCPs (1,174)	Medications used in primary care	PSR visits (self-report); Attendance at pharmaceutical sponsored meetings (self-report)	Quality use of medicine score
	Jones [70]	UK 1995–1997	PCPs^d^	Nine new drugs	Journal advertisements	Prescribing data for the advertised drugs
	Caudill [71]	US 1996	PCPs (446)	Medications for acute bronchitis, hypertension and urinary tract infection	PSR visits (frequency of use)	Cost of prescribing
	Berings [72]	Belgium date not stated	PCPs (128)	Benzodiazepines	PSR visits (*n* visits in last 4 wk)	Prescription of benzodiazepines
	Lurie [73]	US 1987–1988	Hospital physicians (240 faculty staff and 131 residents)	Hospital medications	PSR visits (<5 min and >5 min)	Change in prescribing habit Addition to hospital formulary
	Healthcare Communications [80]	US 1987–1988	Physicians (1184)	Newly promoted medications	Journal advertisements (awareness of)	Market share
	Peay [33]	Australia 1981	PCPs (74) and specialists (50)	Temazepam	PSR visits (contact versus no contact); Direct mailing; Attendance at PSR-sponsored function	Temazepam prescription
	Blondeel [81]	Belgium 1987	PCPs (358)	Medications prescribed by PCPs	PSR visits	Response to 8 simulated patients where prescribing was not advisable. Quality index compiled based on GP medication choices by expert panel (range 1–100) Proneness to prescribe (proxy for prescribing frequency)
	Haayer [74]	Netherlands 1979	PCPs (116)	Medications that would result from 8 case-histories devised by a panel	PSR visits; Journal advertisements; Companies' mailings	Prescribing rationality based on a composite scale (drug choice, duration, dose and use of combination products)
	Walton [75]	US 1976–77	PCPs (29%) and specialists (71%) (1,000 total)	186 different medications	Journal advertisements	Prescriptions of advertised drugs (intention to prescribe)
	Dajda [76]	UK 1975	PCPs in UK^d^	Branded advertised drugs in the UK	Mailed advertisements (number in 1 y)	*n* prescriptions
	Becker [77]	US 1970	PCPs (29), internists (3). osteopathic physicians (5)	Chloramphenicol, equagesic, vitamin B12, methylphenidate, oral contraceptives	Use of journal advertisements PSR visits (frequency)	Proportion of chloramphenicol scripts. Physicians' self-reported prescribing behaviour.

a Experimental partnerships between pharmaceutical company and health authority or academic department.

b Data from pharmaceutical company.

c Information from a market research company.

d Total number unknown.

e Using national prescribing data.

PCP, primary care provider; RCT, randomized controlled trial.

### Pharmaceutical Company Information and Prescribing Quality

Prescribing quality was measured by ten studies with 14 units of analysis [37],[39],[58],[59],[61],[64],[74],[77],[81],[82] (Table 7). Quality was assessed in four distinct ways: quality scoring of prescribing decisions, guideline adherence, prescribing appropriateness of an individual drug class, and prescribing range. Three studies used quality scores calculated by coding physicians' drug choices in responses to clinical vignettes [74],[81],[82]. One of these used an expert panel to derive a quality index (1–100) judging primary care providers' prescribing in response to both their actual prescribing and clinical vignettes [81]. In the latter study learning about the drug first from pharmaceutical sales representatives was associated with lower quality of actual prescribing but the number of pharmaceutical sales representatives' visits was not. There was no significant association between primary care providers seeing more pharmaceutical sales representatives or first learning about the drug from pharmaceutical sales representatives and lower quality responses to case vignettes [81]. Another study combined scales examining indication, effectiveness, safety, dosage, duration, and polypharmacy to produce a seven-point scale measuring rationality of prescribing [74]. Primary care providers' self-reported reliance on pharmaceutical companies for information was associated with lower quality scores [74]. A third study used a quality score for a hypertension scenario where thiazides were considered very appropriate and all other drug groups were considered very inappropriate [82]. Self-reported rates of attendance at pharmaceutical company-sponsored meetings were associated with slightly lower quality scores, but self-reported rates of pharmaceutical sales representative visits had no significant association [82].

**Table 7:** Relationship between exposure to information from drug companies and prescribing quality (by year of publication and then study design/size).

Exposure to Information from Drug Company	Study (First Author Name)	Result in Exposed Group Versus Controls (Where Applicable)	Change in Prescribing Quality Result
**Effect of PSR visits**	de Bakker [61]	Wider prescribing range was associated with more visits from PSRs in the last 4 wk	Beta coefficient +0.18 (*p*<0.05)^a^
	Muijrers [64]	More frequent visits from PSRs was associated with less adherence to prescription guidelines	Multiple linear regression:
			Beta −0.23 (95% CI −0.32 to −0.15) *p*<0.05
	Mansfield [82]	Frequency of visits from PSRs was not associated with a difference in quality score	Pearson coefficient of 0.0363; *p* = 0.247
	Blondeel [81]	Based on responses to 8 case histories:	Multivariate regression analysis:
		First contact with a drug from the pharmaceutical industry was not associated with quality index;	*p*>0.1
		*n* PSRs received was associated with poorer quality index;	*p*>0.05
		Based on prescriptions for actual patients: First contact with a drug from the pharmaceutical industry was associated with reduced quality of prescribing;	*p*<0.01
		*n* PSRs received was not associated with poorer quality index	*p*>0.1
	Becker [77]	Fewer visits from PSRs/month were not associated with a change in the appropriateness of prescribing	Gamma statistic; 0.04, not statistically significant
**Attendance at pharmaceutical sponsored meeting**	Mansfield [82]	Attendance at pharmaceutical sponsored meetings was associated with lower quality scores	Pearson correlation coefficient of 0.0635; *p* = 0.043
	Spingarn [39]	Attendees at a sponsored talk about Lyme disease were less likely to choose appropriate oral antibiotics for mild Lyme disease than nonattendees	0% of attendees (*n* = 22) chose appropriate antibiotics compared to 21% (*n* = 53) of nonattendees; Fisher exact test: *p* = 0.027
		For attendees and nonattendees of a sponsored talk about Lyme disease there was no difference in choice of acceptable treatment for Lyme disease with central nervous system signs	OR = 3.2 (95% CI 0.8–19.2)
		Attendees of a sponsored talk about Lyme disease were more likely to appropriately choose the sponsoring company's treatment for Lyme disease complicated by 2nd degree heart block	OR = 7.9 (95% CI 2.4–29.3)
**Journal advertisements**	Becker [77]	Infrequent use of journal ads as a source of prescribing information by doctors was not associated with a change in the appropriateness of prescribing	Gamma statistic 0.373, not statistically significant
**Total promotional investment/summated scores of commercial information use/general use of commercial sources**	de Bakker [61]	There was a positive correlation for how frequently doctors used the pharmaceutical industry as a source of information and the range of drugs they prescribed	Beta coefficient +0.15 (*p*<0.05)^a^
	Haayer [74]	Frequency of use of information from the pharmaceutical industry was associated with less rational prescribing	Beta coefficient +0.134 ; *p*<0.001
**Information delivered without conventional promotion**	Andersen [37]	Participation in a randomized controlled trial was not associated with a change in guideline adherence at 2 y for trial sponsor's medication	OR 1.00 (95% CI 0.84–1.19)
	Kazmierczak [59]	Mailed warning letters regarding tramadol for those with a seizure risk were not associated with a change in prescription rates for tramadol	9 (10%) prescriptions before and 7 (9%) after warning letters were sent out no association detected
	Soumerai [58]	PSR visits: Propoxyphene use continued a preexisting decline of about 8% a year during the time when warnings from the manufacturing pharmaceutical company were conveyed by PSRs after which time this decline halted, however a statistical association was not shown. Refill rates and rates of overdose did not change following the warnings	No association detected
		Mailed Information: Propoxyphene use continued a preexisting decline of about 8% a year during the time when warnings from the manufacturing pharmaceutical company were expressed by PSRs after which time this decline halted, however a statistical association was not shown. Refill rates and rates of overdose did not change following the warnings	No association detected

a Assumes a wide prescribing range is lower quality prescribing than a narrow prescribing range.

Residents attending a sponsored meeting were more likely than nonattending residents at the same hospital to prescribe the sponsoring company's medication, both when it was appropriate according to the authors and when it was not [39].

Primary care providers who saw more pharmaceutical sales representatives and those who used the pharmaceutical industry in general as a source of information prescribed a wider range of drugs [61]. The authors suggested that this was a sign of lower prescribing quality in the context of recommendations that primary care providers use a limited list of drugs they know well [61].

Two studies measured guideline adherence. One found less adherence by primary care providers who received more frequent visits from pharmaceutical sales representatives [64], while the other found no change in adherence by primary care providers participating in a clinical trial sponsored by a pharmaceutical company [37].

One study of warnings conveyed by pharmaceutical sales representatives and mailed information [58], one of mailed warnings alone [59], and one of representatives' visits and advertisements [77] found that there was no alteration in overall rates of prescriptions judged to be inappropriate.

### Pharmaceutical Company Information and Prescribing Frequency

51 studies [25]–[54],[56]–[60],[62],[63],[65],[67]–[70],[72],[73],[75]–[81] measured prescribing frequency as market share, intention to prescribe, prescription sales, formulary requests, as well as number of prescriptions (63 units of analysis) (Table 8). Below we report separately the results of studies of information delivered with versus without conventional promotion. Within both groups there was one unit of analysis per study.

**Table 8:** Relationship between exposure to information from drug companies and prescribing frequency (by year of publication and then study design/size).

Exposure to Information from Drug Company	Study (First Author Name)	Results	Change in Prescribing Frequency Results
**Effect of PSR visits**	Ching [78]	Higher levels of detailing for enalapril/hydrochlorothiazide and lisinopril/hydrochlorothiazide was associated with higher levels of demand (prescriptions)	Detailing elasticity 0.1–0.27 (*p*<0.05)
	Kreyenbuhl [31]	Meeting PSRs >4 times in the preceding month was not associated with the “add” rather than “switch” strategy for antipsychotic medication prescribing	OR 1.22 (95% CI 0.68–2.20)
	Steinman [8]	PSR visits of ≤5 min versus >5 min were not associated with intention to prescribe	No association detected
		PSR visits to doctors in a small group were associated with increase in more frequent intention to prescribe^a^	OR 12.9 (95% CI 1.2–138.8)^b^
		PSR visits were associated with increased gabapentin prescribing if physician's previous gabapentin prescribing was nil^a^	OR 15.1 (95% CI 3.9–58.2)^b^ reference group - medium prescribers of gabapentin
		PSR visits were associated with increased gabapentin prescribing if physician's previous gabapentin prescribing was low^a^	OR 8.6 (95% CI 2.4–31.4)^b^ reference group, medium prescribers of gabapentin
	Venkataraman [40]	PSR visits were associated with increased *n* prescriptions	Beta coefficient: +0.944 (significant with a 95% CI)
	Canli [32]	PSR visits were associated with increased antibiotic prescribing^a^	*p* = 0.0001*
	Chintagunta [42]	Higher levels of detailing were associated with higher market share for that brand in the three of the countries studied and no significant difference in two others	Detailing related change in market share; US; beta coefficient +0.06; *t* statistic 3 (*p*<0.05); Germany; beta coefficient +0.73; *t* statistic 3.6 (*p*<0.05); France; beta coefficient +4.17; *t* statistic 7.87 (*p*<0.05); Italy; beta coefficient +0.24; *t* statistic 0.96 (*p*>0.05); UK; beta coefficient +0.29; *t* statistic 1.61 (*p*>0.05)
	Narayanan [43]	PSR visits were associated with an increase in market share	1% increase in expenditure on detailing was associated with increases in market shares for promoted drugs ranging from 0.11% to 0.14% (*p*<0.05)
	Verdoux [63]	PSR visits were associated with general practitioners initiating a newer antipsychotic medication	OR 2.80 (95% CI 2.09–3.76); *p* = 0.0001
	Mizik [45]	PSR visits were associated with increased prescribing of Drugs A, B. and C	Drug A: 1 PSR visit generates 1.56 new prescriptions (95% CI 0.8–2.23) or 0.64 visits to induce one prescription
			Drug B: 1 PSR visit generates 0.32 new prescriptions (95% CI 0.22–0.43) or 3.11 visits to induce one prescription
			Drug C: 1 PSR visit generates 0.153 new prescriptions (95% CI 0.11–0.2) or 6.54 visits to induce one prescription
	Donohue [44]	Expenditure on PSR visits is associated with higher probability that the detailed antidepressant is prescribed	Beta coefficient +0.703 (*p*<0.001)
	Stafford [56]	Decreasing promotional expenditure was associated with a decrease in prescribing for alpha blockers^c^	Decreased with decreased promotion
	Manchanda [46]	PSR visits were associated with more new prescriptions	1.8 detailing visits results in 5 new prescriptions (average result)^b^
	Manchanda and Chintagunta [47]	PSR visits were not associated with a significant change in mean prescriptions	Beta coefficient +0.83 detailing *t* statistic 0.675 (*p*>0.05)
		More frequent PSR visits were associated with diminishing increases in prescribing	Quadratic term for PSR visits: −0.49; *t* statistic −0.49 (*p*>0.05)
	Berndt [48]	PSR detailing were associated with increased cumulative days of therapy	Beta coefficient +0.7414; *t* statistic 43.12 (*p*<0.01)
	Rosenthal [79]	PSR visits were associated with increased frequency of prescription	Beta coefficient +0.017; *t* statistic 4.25 (*p*<0.05)
	Prosser [67]	PSR visits were more likely to be cited as a prescribing influence by high prescribers than by low prescribers	OR 7.32 (95% 1.64–32.61); Fisher exact test; *p* = 0.002
	Azoulay [49]	PSR detailing is associated with diffusion of product information and performance on the product market with marketing activities having a more pronounced effect than scientific information in the form of clinical trial reports^c^	Beta coefficient +0.654; *t* statistic 10.17 (*p*<0.05)
	Gonul [69]	PSR visits in minutes were a positive predictor of medication prescription	Beta coefficient +0.1085; *t* statistic 5.32 (*p*<0.001)
	Caamano [68]	PSR visits were not associated with the *n* prescriptions	Adjusted regression coefficient −0.490.001; *p* = 0.998
	Schwartz [27]	PSR visits to residents were associated with increased initiation of prescriptions for 12 drugs^a^	*p*<0.05 for all*
		PSR visits were not associated with increased prescription of one medication however for this medication unlike the others there had been more PSR visits in the control group	No association detected (*p*>0.05)*
	Rizzo [50]	PSR visits were associated with increased prescription sales	Beta coefficient +0.28; *t* statistic 4.19 (*p*<0.01)
		PSR visits may result in diminishing returns given the quadratic beta coefficient is statistically significant and negative	Quadratic sales coefficient for PSR visits: −0.490.01 (*p*>0.05)
	Chren [38]	PSR meetings were associated with a formulary request	Multivariate result: OR = 3.4 (95% CI 1.8–6.6); *p*<0.001
	Berings [72]	PSR visits were not significantly associated with benzodiazepine prescribing^a^	Linear regression analysis: beta 0.16 (*p* = 0.05 to 0.1)
	Cleary [26]	PSR visits were associated with an increase in prescribing of promoted medications; prescribing of them decreased when they were not promoted	Ceftriaxone 24.2% and 27.8% increase in promoted periods; *p*<0.05
			Cefotaxime 14.6% and 26.2% increase in promoted periods; *p*<0.05
			Ceftazidime (promoted in period I but not promoted in period II): 27.7% decrease when not promoted in period II (*p*<0.05) and 10% increase in period III after being promoted again; *p*<0.05
	Lurie [73]	PSR visits for faculty staff for less than 5 min were associated with more prescribing	Logistic regression coefficient 0.016; *p* = 0.03
		PSR visits for faculty staff for more than 5 min were not associated with a change in prescribing	*p*>0.10 (coefficient not presented where result not significant)
		PSR visits for faculty staff for less than 5 min were not associated with an addition to the hospital formulary	Logistic regression coefficient 0.014; *p* = 0.06
		PSR visits for faculty staff for more than 5 min were not associated with an addition to the hospital formulary	*p*>0.10 (coefficient not presented where result not significant)
		PSR visits for residents for less than 5 min were associated with more prescribing	Logistic regression coefficient 0.049; *p* = 0.003
		PSR visits for residents for more than 5 min were not associated with a change in prescribing	*p*>0.10 (coefficient not presented where result not significant)
		PSR visits for residents for less than 5 min were not associated with an addition to the hospital formulary	*p*>0.10 (coefficient not presented where result not significant)
		PSR visits for residents for more than 5 min were not associated with an addition to the hospital formulary	*p*>0.10 (coefficient not presented where result not significant)
	Peay [33]	PSR visits were associated with temazepam prescription	Multivariate regression: −0.35 (*p*<0.002)
	Blondeel [81]	Based on responses to 8 case-histories:	Multivariate regression:
		First contact with a drug from the pharmaceutical industry was not associated with proneness to prescribe	*p* = 0.05–0.1
		Number of PSRs received was not associated with proneness to prescribe	*p*>0.1
		Based on prescriptions for actual patients:	
		First contact with a drug from the pharmaceutical industry was not associated with proneness to prescribe	*p*>0.1
		Number of PSRs received was associated with proneness to prescribe	*p*<0.05
	Mackowiak [52]	PSR visit expenditure was not associated with a change market size nor market share for benzodiazepines or diuretics	No association detected
	Becker [77]	PSR visits per month were not associated with chloramphenicol prescribing	Gamma statistic 0.236; not significant
**Journal advertisements**	Hemminki [25]	Journal advertisements were associated with a trend for increased hormone replacement therapy (HRT) prescribing in Estonia	Increased prescriptions
	Charbit [34]	Journal advertising was associated with increased prescriptions of ARA. When journal advertisements for ACE inhibitors and CCB decreased, their market share also decreased	10.5% decrease in mean annual advertising of ACE inhibitors associated with 19.3% decrease in market share 11% decrease in mean annual advertising for CCBs associated with 19.3% decrease in market share. 20.5% increase in mean annual advertising rate for ARAs associated with 22.9% increase in market share
	Prosser [67]	Journal advertisements were no more likely to be cited as a prescribing influence by high prescribers than by low prescribers	9% high prescribers versus 0% of low prescribers; Fisher exact test; *p* = 0.18
	Azoulay [49]	Journal advertisements were associated with diffusion of product information and performance on the product market with marketing activities having a more pronounced effect than scientific information in the form of clinical trial reports^c^	Beta coefficient +0.112; *t* statistic 4.753 (*p*<0.05)
	Jones [70]	Journal advertisements were not associated with PCP prescribing	No association detected
	Healthcare Communications [80]	Journal advertisement recognition was associated with increased market share for the advertised medication	14.5% difference in market share between those physicians not recognising advertisements (19.6%) and those associating the advertisement message with the product (34.1%)
	Walton [75]	Journal advertisement recognition was associated with medication prescription	OR 1.68 (95% CI 1.21–2.35)^b^
	Becker [77]	Infrequent use of journal advertisement use was not associated with chloramphenicol prescribing	Gamma statistic −0.186 not statistically significant
		Infrequent use of journal advertisements to learn about the usefulness of new medications was associated with reduced chloramphicol prescribing^a^	Gamma statistic +0.51; *p*<0.05
**Attendance at pharmaceutical company-sponsored meeting**	Kreyenbuhl [31]	Attendance at pharmaceutical sponsored CME meetings more than once in the preceding month was associated with the “add” rather than “switch” strategy for antipsychotic medication prescribing^a^	OR 2.32 (95% CI 1.29–4.18); *p* = 0.005*
	Venkataraman [40]	Attendance at pharmaceutical sponsored meetings was not significantly associated with prescriptions for 7 out of 12 brands	Beta coefficient −0.659 (significant with a 90% CI)
	Narayanan [43]	Attendance at pharmaceutical company-sponsored meetings was associated with an increase in promoted medication market share	A 1% increase in expenditure on “other marketing activities” (including meetings) was associated with increases in market shares for promoted drugs ranging from 0.02% to 0.04% (*p*<0.05)
	Huang [65]	Attendance at pharmaceutical sponsored conferences was associated with more prescriptions of the corresponding sponsored antidepressant^a^	Pearson correlation coefficient; 2001–2002: 0.87; *p*<0.01, 2002–2003: 0.73; *p*<0.01
	Spingarn [39]	Attendance at a pharmaceutical sponsored meeting was not associated with the intention to prescribe the promoted medication where it was indicated	OR 2.51 (95% CI 0.91–6.95)
	Orlowski [28]	Attendance at pharmaceutical sponsored meeting was associated with more prescriptions of medications being discussed	Drug A: 81 (±44) prescriptions before, 272 (±117) prescriptions after; *p*<0.001 (Wilcoxon rank sum)
			Drug B: 34 (±30) prescriptions before, 87 (±24) prescriptions after; *p*<0.001 (Wilcoxon rank sum)
	Bowman [60]	Attendance at pharmaceutical sponsored courses was associated with more prescriptions of medication made by sponsoring company	Before and 6 mo after 3 sponsored course involving sponsoring company's drugs:
			Course I: Nifedipine, increase in prescriptions 5.6%; *p*<0.05*
			Course II: Metoprolol, increase in prescriptions 12.4%; *p*<0.05*
			Course III: Diltiazem, increase in prescriptions 18.7%; *p*<0.05*
	Peay [33]	Attendance at pharmaceutical sponsored meeting was not associated with prescription of temazepam	No association detected
**Mailed information from pharmaceutical companies**	Prosser [67]	Mailed information was no more likely to be cited as an influence by high prescribers than low prescribers^a^	9% for high prescribers, 0% for low prescribers; Fisher exact test; *p* = 0.18
	Peay [33]	Mailed information was not associated with a change in temazepam prescribing frequency	No association detected
	Dajda [76]	Mailed advertisements to general practitioners was associated with an increase in prescriptions	Correlation coefficient 0.08
**Advertising on clinical software**	Henderson [29]	Advertisements on clinical software were not associated with a difference in prescribing for all advertised medications combined	Adjusted OR 0.96 (95% CI 0.87–1.06); *p* = 0.42
**Total promotional investment/summated scores of commercial information use/general use of commercial sources**	Greving [30]	Commercial information sources of information were associated with an increase in rates of prescribing of angiotensin receptor blocking medications	OR 2.0 (95% CI 1.5–2.6)
		Commercial information sources of information were not associated with an increase in the *n* doctors prescribing angiotensin receptor blocking medications	OR 12.8 (95% CI 0.20–816.58)
	Windmeijer [41]	Expenditure on pharmaceutical promotion was associated with more prescribing	Beta coefficient +0.0137; *t* statistic 2.98 (*p*<0.01)
	Auvray [57]	Total promotional investment was associated with an increase in the *n* prescriptions	No statistical measures presented
	Peay [33]	Commercial information sources were associated with a preference for temazepam prescribing^a^	*p*<0.036 (*t* test)
		Commercial information sources were associated with earlier temazepam prescribing^a^	*p*<0.045 (*t* test)
	Hurwitz [51]	Promotion of the branded leading drug was associated with increased market share especially for acute or sporadic conditions	Beta coefficient +0.295; *t* statistic 4.34 (*p*<0.01)
		Promotion of “following generic drugs” was associated with reduced the market share for the leading drug	Beta coefficient −0.150; *t* statistic 2.14 (*p*<0.05)
	Mackowiak [52]	Expenditure on PSRs and journal advertisements was not associated with a change in market size nor market share for benzodiazepines or diuretics	No association detected
	Leffler [53]	The promotional intensity for new products was not associated with increased market share for the entrant product 2 y post introduction	Beta coefficient +0.88; *t* statistic 1.89, *p*>0.05
		The promotional intensity for new products introduced over a 9-y period was associated with increased market share for the entrant products	Beta coefficient +1.25; *t* statistic 2.35, *p*<0.05
	Telser [54]	Overall promotional intensity was associated with the market share of entrant drugs in the hospital and drug store market in the period 1964–1968	Drug store: beta coefficient +1.28; *t* statistic +2.20 (*p*<0.05)
			Hospital: beta coefficient +1.45; *t* statistic +2.61 (*p*<0.05)
		Overall promotional intensity was not associated with the market share of entrant drugs in the hospital and drug market in the period 1968–1972	Drug store: beta coefficient +1.19; *t* statistic +0.60 (*p*>0.05)
			Hospital: beta coefficient +0.608; *t* statistic +1.20 (*p*>0.05)
**Information delivered without conventional promotion**	Andersen [37]	Participation in pharmaceutical funded research was associated with increase in the sponsoring company's share of asthma drug in practices conducting the trial compared to control practices	6.7% increase (95% CI 3.0%–11.7%)^b^
	Freemantle [35]	PSR visits were not associated with an increase in the prescription of the detailed medication	OR = 1.04 (95% CI 0.83–1.31); *p* = 0.73
	Dolovich [36]	PSR visits were not associated with a change in the market share of amoxicillin	Intervention group: +0.63% market share, control group: −0.72% market share; *p* = 0.15
	Kazmierczak [59]	Mailed warning letters regarding tramadol for those with a seizure risk were not associated with a change in prescription rates for tramadol^a^	Before mailing: 10% prescribing rate, after mailing 9% prescribing rate.
	Soumerai [58]	PSR visits: Propoxyphene use continued a preexisting decline of about 8% a year during the time when warnings from the manufacturing pharmaceutical company were expressed by PSRs after which time this decline halted, however a statistical association was not shown. Refill rates and rates of overdose did not change following the warnings^a^	No association detected
		Mailed information: Propoxyphene use continued a preexisting decline of about 8% a year during the time when warnings from the manufacturing pharmaceutical company were expressed by PSRs after which time this decline halted, however a statistical association was not shown. Refill rates and rates of overdose did not change following the	No association detected

a Study authors reported that exposure to information from drug companies was associated with decreased quality of prescribing.

b Reported by study authors as statistically significant.

c Study authors reported that exposure to information from drug companies was associated with increased quality of prescribing.

*Chi-squared statistic.

ACE, angiotensin converting enzyme; ARA, angiotensin receptor antagonist; CCB, calcium channel blocker; CME, continuing medical education.

### Conventional Promotional Techniques

#### Pharmaceutical sales representative visits

Of the 29 studies of pharmaceutical sales representative visits, 17 found only an association with increased prescribing of the promoted drug [26],[32],[33],[38],[40],[43]–[50],[63],[67],[78],[79]. None found less frequent prescribing. Of the remaining 11, six studies had mixed results: finding a significant association with more frequent prescribing for some measures but no significant association for others [27],[42],[62],[69],[73],[81]. Five did not detect any significant relationship [31],[52],[68],[72],[77]. One study did not use statistical tests for associations. It found that during the time that spending by pharmaceutical companies on promotion of a medication dropped to zero, there was also a significant drop in prescribing of that medication. However most of the decreases in promotion and prescribing occurred after the publication of evidence of problems with that medication [56].

Nine of these studies with either positive or mixed results provided insights into features of pharmaceutical sales representative visits that modified the impact of these visits on prescribing [40],[46],[49],[62],[67],[69],[73],[78]. An association with more frequent prescribing was more likely when pharmaceutical sales representatives visited groups of physicians, when physicians had lower baseline prescribing of the promoted drug [62], and when physicians had larger prescribing volumes overall [67]. Longer pharmaceutical sales representative visits to physicians and residents were also more likely to be associated with increased prescribing [69],[73]. More frequent pharmaceutical sales representative visits were associated with diminishing returns [46],[50],[69].

In addition to increasing the promoted drug's market share, pharmaceutical sales representative visits were associated with a decrease in the market share of competitor products [78]. Pharmaceutical sales representative visits were more likely to be associated with more frequent prescriptions for drugs judged more effective and also for drugs with more side effects [40]. However the authors of that study did not attempt to measure whether higher levels of use represented a change in prescribing quality. Another study found that pharmaceutical sales representative visits were associated with a greater increase in market share for new entrants into a therapeutic field than was positive scientific information [49].

#### Journal advertisements

Four out of the eight studies measuring the effects of journal advertisements presented data but did not include statistical tests [25],[34],[70],[80]. One of these noted use of a medication class increased after pharmaceutical advertising commenced in a country where the medication class was previously available but was not promoted [25]. One study visually compared graphs of the monthly number of advertisements and prescriptions for a group of nine drugs and found no clear relationship between the extent of the advertising of a drug and the amount of prescribing by general practitioners [70]. One study found that the market share of a medication was higher amongst physicians who recognised the advertisement for that medication compared to those who did not [80]. The last study observed decreased prescribing of two drug classes at the same time that advertising decreased [34].

Of the four studies that included statistical tests, one found that journal advertisements have a more pronounced effect on market share for the advertised drug than does positive scientific information published in medical journals [49]. A cross-sectional study found contradictory results. Self-reported infrequent use of journal advertisements by physicians to learn about new medications was not associated with frequency of prescribing. However, infrequent use of journal advertisements was associated with less chloramphenicol prescribing [77]. One cross-sectional study found that physicians who recalled advertisements became prescribers of the advertised products in consistently larger proportions than those who did not recall advertisements [75]. Another study found that 9% of high prescribers of new drugs cited advertisements as an influence on their prescribing compared to 0% for low prescribers; however, this was not a statistically significant association [67].

#### Attendance at pharmaceutical company-sponsored meetings

There were eight studies of pharmaceutical company-sponsored meetings. Five found positive associations with prescribing frequency [28],[31],[43],[60],[65]. Three studies did not detect a significant association [33],[39],[40].

#### Mailed information from pharmaceutical companies

One of the three studies of mailed promotional material found an association with increased prescribing [76]. The others found no association [33],[67].

#### Advertising in clinical software

A single study examined the effect of advertising in clinical practice software and found no association with prescribing frequency for six medications and less prescribing of one medication [29]. The overall result was no association between advertising and prescribing frequency.

#### Total promotional investment

Eight studies combined the outcome measures for various exposures to pharmaceutical company information or measured overall promotional investment, a proxy for the amount of exposure to information from pharmaceutical companies. Three studies found that total promotional investment was positively associated with prescribing frequency [30],[33],[51]. Two studies found both positive results and no association [53],[54]. One study did not detect an association [52].

#### Meta-analysis of promotional information and prescribing frequency

**Figure 2:**
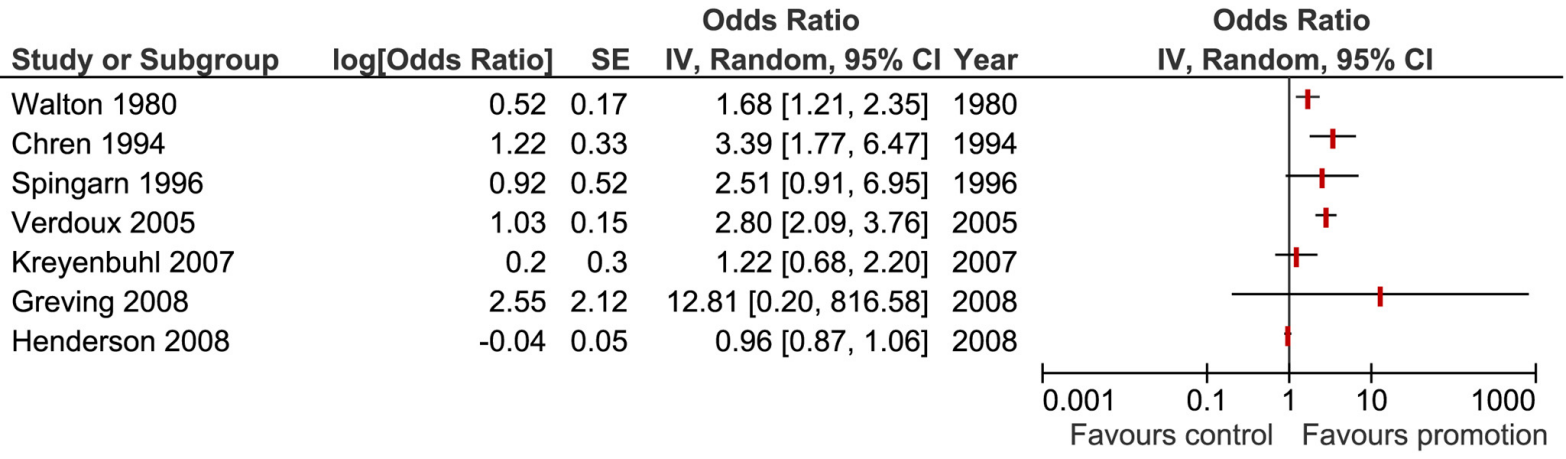
Forest plot displaying the effect of promotional information on physicians' prescribing of the promoted medication.

We pooled results from a total of seven studies using a random effects model to examine whether exposure to promotion was associated with prescribing of the promoted medication. The seven study results included in the meta-analysis showed significant heterogeneity (*I* ^2^ = 91% [95% confidence interval (CI) 84%–95%], tau^2^ = 0.35), and therefore we have presented the forest plot without the pooled outcome (Figure 2) [29],[30],[31],[38],[39],[63],[75]. Using sensitivity analysis we found that study design, quality factors, year of publication, and type of physician did not explain this heterogeneity. One study provided two units of analysis with outcomes amenable to meta-analysis: a significant association for attendance at sponsored meetings and a nonsignificant result for pharmaceutical sales representative (PSR) visits [31]. We included only that nonsignificant result in the forest plot (Figure 2). When meta-analysis was conducted using the significantly positive result for attendance at a pharmaceutical company-sponsored meeting, the summary result and level of heterogeneity did not differ greatly. The largest difference detected was between exposure to active promotional information (OR 2.34, 95% CI 1.50–3.65), (*I* ^2^ = 59%, 95% CI 0%–86%, tau squared = 0.11) [31],[38],[39],[63] and passive promotional information (OR 1.24, 95% CI 0.72–2.15) (I^2^ = 89.5%, tau squared = 0.14) [29],[75].

### Information Delivered Without Conventional Promotion Techniques

Five studies looked for associations between information delivered without conventional promotion techniques and the frequency of physicians' prescribing [35],[36],[37],[58],[59]. One randomized controlled trial partnered a local health authority and a pharmaceutical company with the aim of promoting a less expensive drug [35], and the other randomized controlled trial aimed to promote rational prescribing through evidence-based detailing by a pharmaceutical company in partnership with an academic institution [36]. Neither found an association with physicians' prescribing. A single controlled-cohort study of a pharmaceutical company-funded randomized controlled trial found that physicians' participation in recruiting subjects was associated with an increase in the number of prescriptions of the sponsoring company's drug [37]. One time-series analysis found no change in the rate of decline in the prescribing of a medication when the main manufacturer was required by a regulatory agency to deliver an educational program warning about problems with the drug via mailed information and pharmaceutical sales representative visits [58]. A cross-sectional study found no change in prescription rates following warning letters regarding drug side effects [59].

### Pharmaceutical Company Information and Prescribing Costs

Eight studies (Table 9) [35],[41],[50],[55],[66],[68],[69],[71] measured prescribing costs as costs per physician, price elasticity, and changes in generic prescribing (ten units of analysis). In the United States, one econometric time-series analysis found that pharmaceutical sales representative visits were associated with increased price sensitivity among physicians prescribing in one therapeutic class [69], and another found the opposite effect for hypertension [50]. A third, more recent, econometric study found that promotional outlay (the total for pharmaceutical sales representative visits, journal advertisements, and direct mail) was associated with reduced price sensitivity for primary care providers and psychiatrists in 11 therapeutic classes consisting of more than 50% of the Dutch drug market [41]. Of three cross-sectional studies, two detected an association between pharmaceutical sales representative visits and higher prescribing costs [66],[71], but one did not detect an association [68]. One study also found that low cost prescribers were more likely to have rarely or never read promotional mail or journal advertisements from pharmaceutical companies than high cost prescribers [66]. One time-series analysis found that reduced exposure to pharmaceutical sales representative visits and promotional material was associated with an increase in generic prescribing [55]. A randomized controlled trial of pharmaceutical sales representative visits in a noncommercial partnership between a pharmaceutical company and a local health authority measured physicians' prescribing costs for the target drug class and found no effect [35].

**Table 9:** Relationship between exposure to information from drug companies and prescribing costs (by year of publication and then study design/size).

Exposure to Information from Drug Company	Study (First Author Name)	Results	Change in Prescribing Costs
**Effect of PSR visits**	Watkins [66]	High cost prescribers were more likely to see PSRs at least once a week than low cost prescribers	OR 3.11 (95% CI 2.48–3.89); *p*<0.01^a^
	Caamano [68]	There was no association between PSR visits and the cost of prescriptions	Adjusted regression coefficient: 21.0; *p* = 0.962
	Gonul [69]	PSR visits were associated with increased physicians' price sensitivity	Maximum likelihood estimate, 0.0012; *t* statistic 3 (*p*<0.001)
	Rizzo [50]	PSR visits were associated with reduced price elasticity for the promoted drug	Sales estimate +0.14; *t* statistic 2.97 (*p*<0.01)
	Caudill [71]	Frequency of PSR visits was associated with higher prescribing costs	Multivariate regression beta +0.155; *p* = 0.01
**Journal advertisements**	Watkins [66]	High cost prescribers were less likely to “rarely or never” read journal advertisements than low cost prescribers	OR 0.79 (95% CI 0.64–0.98); *p* = 0.02^a^
**Mailed information from pharmaceutical companies**		High cost prescribers were less likely to “rarely or never” read mailed information than low cost prescribers	OR 0.49 (95% CI 0.38–0.64); *p*<0.01^a^
**Total promotional investment/summated scores of commercial information use/general use of commercial sources**	Spurling [55]	Reduced *n* PSR visits and volume of promotional material were associated with an increased generic prescribing at 3 and 9 mo	3 mo: OR 2.28 (95% CI 1.31–3.86); *p* = 0.0027^a^
			9 mo: OR 2.07 (95% CI 1.13–3.82); *p* = 0.016^a^
	Windmeijer [41]	Promotional outlay (PSR visits, journal advertisements, direct mail) was associated with reduced price elasticity for promoted drugs	ln regression coefficient −0.0102 (se 0.0055) *p*<0.05
**Information delivered without conventional promotion**	Freemantle [35]	There was no significant difference in costs between the group that was detailed by PSRs instructed by a local health authority and the control group	Mean difference: £122.32 (95% CI −£94.91 to £342.91)

a Chi-squared statistic.

## Discussion

### Overview

We found that the reported relationship between exposure to information provided directly by pharmaceutical companies and the quality, frequency, and cost of prescribing varied from case to case. However, with only one exception [39], the included studies reported that exposure to information from pharmaceutical companies was associated with either lower prescribing quality or no association was detected. Similarly, exposure to information from pharmaceutical companies was associated with either an increase in prescribing frequency or no association was detected. Three studies found that exposure was associated with increased drug sales up to a point of diminishing returns beyond which more promotion was increasingly less effective [46],[50],[69]. Finally, with only one exception [69], exposure to information from pharmaceutical companies was associated with an increase in prescribing costs or no association was detected.

This review has supported, updated, and extended the findings of previous reviews regarding the effects of exposure to information from pharmaceutical companies. 38 of the 58 included studies (66%) were not included in previous systematic reviews on this topic [25],[29]–[32],[34],[35],[40]–[42],[44],[48],[49],[51]–[59],[61]–[68],[70],[72],[75],[76],[78]–[82], including seven of the ten studies of prescribing quality [37],[58],[59],[61],[64],[81],[82] and four of the seven studies of prescribing costs [35],[55],[66],[68].

Most of the included studies measured the frequency of prescribing. Amongst these, the studies of informational exposure where physicians are active participants, such as representatives' visits, sponsored meetings, or sponsored trials, more consistently found associations with higher prescribing frequency than studies of more passive exposures, such as journal advertisements and mailed information. Poor study quality precludes confident conclusions about journal advertising. However, one higher quality econometric analysis found that advertisements in journals were associated with a more pronounced effect on market share than positive scientific findings published in journals [49]. Also there are claims in the marketing literature that the relatively low cost of passive methods and their ability to synergistically increase the effectiveness of active methods makes them cost effective components of sales campaigns [84].

### Limitations of Included Studies

All of the included studies had design limitations (Tables 1–5). We found only two randomized controlled trials [35],[36]. Both lacked adequate reporting of allocation concealment and blinding. These two trials did not examine standard promotional practice but instead assessed novel partnerships of government or academia with industry aiming for less expensive, higher quality prescribing. On the basis of these two negative randomized controlled trials, it seems unlikely that similar partnerships will have beneficial effects on prescribing. No definite conclusions can be extrapolated from these studies to standard promotional practice.

All other included studies were observational and thus able to measure associations but not prove causation. There is a risk that reported associations may be false positives, and that statistically significant findings may not necessarily be clinically significant. One example is the study by Mizik et al. that reports only a small increase in prescriptions associated with visits from pharmaceutical sales representatives [45]. Associations may also arise from confounding, bias, or chance. False negatives or inaccurate estimation of effect sizes may result from small sample sizes, measurement errors, overly complex models, or “contamination” when prescribers who are thought to be unexposed are actually influenced by other methods. For example in a study of promotional meetings, nonattenders may be influenced by sales representatives thus reducing the difference from attenders in their prescribing. Another possible source of contamination is indirect influence by colleagues who have been influenced directly.

To the extent that the measured associations are real, causality may be bidirectional. The influence of information from pharmaceutical companies on prescribing is a likely explanation for the associations given that the major purpose of pharmaceutical promotion is to influence prescribing [3]. However, it is also possible that physicians who prescribe larger quantities, more expensively or less appropriately may allow themselves to be exposed to, or attract, more promotional information.

Some studies found no association between exposure to information from pharmaceutical companies and prescribing outcomes or small effect sizes that seem unlikely to be clinically significant. Some of these may be false negatives or underestimations caused by study flaws, but it is likely that information from companies sometimes has little or no effect, especially when the information is not designed to increase sales, e.g., letters warning about safety problems. Most of the studies included in this review examined single components of promotional campaigns that may have little or no effect alone but have a synergistic effect in combination with other components. Promotion may be less effective if it is used beyond the point of diminishing returns or is up against similarly effective promotion for another similar product.

Given the controversial nature of this topic, there are many reasons why the studies could be biased overall in either direction. Authors may have produced results consistent with their ideological bias. Also reciprocal obligation to funders who preferred certain results may have lead to bias with or without conscious awareness. Publication and outcome reporting bias may have led to underrepresentation of negative, positive, uninteresting, or unwanted findings.

### Strengths and Weaknesses

The strengths of this review include use of a comprehensive search strategy over multiple databases without any language exclusions. We consulted widely with experts in the field and we used validated instruments to assess quality of the studies. However, only one of the included studies was conducted in a low-income economy, as defined by the World Bank, so the effects of promotion there are less certain [33]. This study found a positive association between pharmaceutical promotion and prescribing frequency. Promotion may be more influential in these countries given the relative paucity of independent sources of information [85],[86].

Our efforts to access data that was not in the databases we searched had mixed results. Messages on e-mail discussion groups and contact with experts yielded five additional studies subsequent to the initial search [34],[43],[80]–[82] whose results were consistent with the entire review. By contrast, pharmaceutical companies did not provide us with any information that was not already in the public domain. However five studies included in this review analyzed confidential data from pharmaceutical companies and their results were also consistent with the review as a whole [33],[35],[37],[40],[46].

Given the wide range of knowledge and experience among the sources that we consulted and the expertise in our group, we are confident that we exhausted all reasonable avenues in our attempt to obtain additional literature.

### Data Interpretation

Of the 58 studies included in this review, 38 studies reported a single unit of analysis with 25 (66%) finding significant associations between exposure to information from pharmaceutical companies and the quality, frequency, and cost of prescribing and eight (21%) finding no associations. The remaining five (13%) had multiple measures and found significant associations on some measures but not on others. The 20 studies with more than one unit of analysis reported 49 units of analysis of which 21 (43%) found significant associations, 24 (49%) found no associations, and four (8%) found mixed results. The difference between the results of the single versus multiple unit of analysis studies is significant (*p*<0.05 Freeman-Halton extension of the Fisher exact test). This difference may have been caused by publication bias against publication of single unit of analysis studies when no association was found. We believe the pattern of results suggests that there was little or no reporting bias for the multiple unit of analysis studies. Because the multiple unit of analysis studies found no association more often than the single unit of analysis studies, multiple mentions of the former studies in our narrative synthesis will not exaggerate the frequency of findings of significant associations.

Interpretation of our meta-analysis requires caution because many studies included in the narrative synthesis could not be included in the meta-analysis. Where a sufficient number of studies could be combined, there was significant heterogeneity. The summary result has not been presented because it is unlikely to accurately reflect the true effect size of most promotional campaigns for two main reasons. First, effect sizes varied widely so it is likely that promotional campaigns often have effect sizes far from average. Second, single promotional techniques are likely to be less effective individually than campaigns employing multiple promotional methods.

A sensitivity analysis found the difference between passive and active promotion is one possible cause of heterogeneity. Other possible explanations for variation in the effectiveness of promotion include variation from campaign to campaign in the relative benefits of the drug being promoted, the promoter's skills and budget, and the target group's level of resistance to promotion.

### Conclusions

The limitations of studies reported in the literature mentioned above mean that we are unable to reach any definitive conclusions about the degree to which information from pharmaceutical companies increases, decreases, or has no effect on the frequency, cost, or quality of prescribing. In theory, advertising may be beneficial in several ways: by distributing information and thus improving the quality of prescribing [20],[78], by reducing costs through increasing price-elasticity [69], by increasing prescribing of drugs that provide better health outcomes, or by improving the cost-effective use of healthcare resources. Because of the limitations of both the included studies and this review we have not disproved those theories but we have found little evidence to support them and have found some evidence of increased costs and decreased quality of prescribing. Any conclusions about harm or benefit for patients are speculative because none of the studies that we found examined clinical outcomes. One clear conclusion from this review is that we did not find evidence of net improvements in prescribing associated with exposure to information from pharmaceutical companies.

Some argue that prescribers have an ethical duty to avoid exposure to pharmaceutical promotion [13],[87]–[89]. Even ineffective promotional information may be harmful if it wastes prescribers' time or if the money spent on promotion increases the cost of medicines [90]; this is of concern given the large expenditure involved [1],[2]. In the absence of evidence of net improvement in prescribing from exposure to promotional information, we recommend that practitioners follow the precautionary principle and thus avoid exposure to information from pharmaceutical companies unless evidence of net benefit emerges.

## Supporting Information

Alternative Language Abstract S1 Malaysian translation of the abstract by NO.
(0.04 MB DOC)

Alternative Language Abstract S2 French translation of the abstract by AIV.
(0.05 MB DOC)

Alternative Language Abstract S3 Spanish translation of the abstract by Diana L. Matallana.
(0.05 MB DOC)

Text S1 MOOSE checklist.
(1.72 MB PDF)

## Abbreviations

CI: confidence interval
OR: odds ratio
PSR: pharmaceutical sales representative
