# Supplementary material for: Information from Pharmaceutical Companies and the Quality, Quantity, and Cost of Physicians' Prescribing: A Systematic Review
Source: PLoS Med. 2010 Oct 19;7(10):e1000352. doi: 10.1371/journal.pmed.1000352 (PMC2957394; doi:10.1371/journal.pmed.1000352)
Supplement: Alternative Language Abstract S1 — Malaysian translation of the abstract by NO. (0.04 MB DOC) [file pmed.1000352.s001.doc]

**Abstrak**

**Latar belakang**

Syarikat farmaseutikal membelanjakan kira-kira USD $ 57.5 billion bagi mempromosikan ubat-ubatan di Amerika Syarikat pada tahun 2004. Pihak industri mendakwa promosi farmaseutikal menyediakan maklumat saintifik dan pendidikan untuk doktor. Walaupun bukti-bukti menunjukkan bahawa promosi farmaseutikal boleh memberikan kesan negatif terhadap corak preskripsi untuk pesakit, para doktor mempunyai pandangan yang berbeza tentang promosi farmaseutikal. Kajian ini bertujuan untuk menilai hubungan antara pendedahan terhadap maklumat dari syarikat farmaseutikal dan kualiti, kuantiti dan kos preskripsi yang dikeluarkan oleh doktor.

**Kaedah dan Penemuan**

Kami telah mencari kajian-kajian yang menyelidik corak preskripsi yang dikeluarkan oleh doktor-doktor yang terdedah kepada maklumat ubat-ubatan dari syarikat farmaseutikal seperti dari eksekutif pemasaran, iklan di jurnal, maklumat yang dihantar kepada doktor, perisian bantuan penentuan preskripsi ubat-ubatan, mesyuarat dan kajian klinikal yang ditaja oleh syarikat farmaseutikal. Indikator yang telah diukur adalah kualiti, kuantiti dan kos preskripsi yang dikeluarkan oleh doktor. Kami telah menjalankan pencarian menggunakan MEDLINE (1966 - Februari 2008), International Pharmaceutical Abstracts (1970-Februari 2008), Embase (1997-Februari 2008), Current Contents (2001-2008) dan Central (The Cochrane Library Issue 3, 2007) dengan menggunakan kata pencarian yang telah ditetapkan dengan bantuan seorang pustakawan yang berpengalaman. Disamping itu, bagi mendapatkan maklumat tersebut, kami juga telah memeriksa senarai rujukan didalam laporan kajian dan menghubungi pakar-pakar didalam bidang ini dan syarikat-syarikat farmaseutikal.

Kajian klinikal secara rawak dan survey yang menilai maklumat ubat-ubatan daripada syarikat farmaseutikal dan corak preskripsi oleh doktor disemak secara bebas oleh dua penulis dengan menumpukan kepada kualiti metodologi kajian-kajian tersebut. Kajian-kajian yang tidak melaporkan hasil kajian mereka secara lengkap tidak dimasukkan dalam senarai kajian bakal yang disemak. Sebanyak 255 laporan kajian yang lengkap diperolehi dari database elektronik (7185 kajian) dan dari sumber-sumber lain (138 kajian). Dari 255 laporan kajian, 179 tidak dimasukkan kedalam senarai untuk di semak secara terperinci kerana tidak memenuhi syarat kelayakan dan 18 kerana faktor kualiti. Diakhir proses semakan, 58 artikel memenuhi syarat kajian dengan 87 unit analisa yang berbeza. Data diambil secara bebas oleh dua penulis dan sintesis secara naratif dilakukan mengikut garispanduan Moose.

Pendedahan terhadap maklumat ubat-ubatan dari dari syarikat farmaseutikal dikaitkan dengan corak preskripsi yang berkualiti rendah atau tidak berkaitan dengan satu pengecualian, frekuensi pengeluaran preskripsi yang lebih tinggi atau tidak berkaitan dan kos preskripsi yang lebih tinggi atau tidak berkaitan dengan satu pengecualian. Sintesis naratif keputusan kajian yang pelbagai disokong oleh meta-analisis kajian-kajian yang meneliti frekuensi pengeluaran preskripsi yang menunjukkan keheterogenan yang signifikan.Limitasi utama kajian ini adalah kebanyakan laporan kajian yang dianalisa adalah bersifat observasional

**Kesimpulan**

Dengan sedikit pengecualian, kajian yang meniliti kesan pendedahan terhadap maklumat yang diberikan secara langsung oleh syarikat farmaseutikal mendapati ia adalah berkait dengan frekuensi pengeluaran preskripsi yang lebih tinggi atau kos preskripsi yang lebih tinggi atau corak preskripsi yang berkualiti rendah atau tiada kaitan yang signifikan. Kami tidak mendapati sebarang bukti dalam pembaikan corak preskripsi tetapi kajian-kajian yang ada tidak menyangkal yang kemungkinan ia boleh diperbaiki. Kami mengesyorkan bahawa pengamal berhati-hati dan mengelakkan pendedahan terhadap maklumat ubat-ubatan yang disampaikan oleh syarikat-syarikat farmaseutikal.
