# Supplementary material for: Information from Pharmaceutical Companies and the Quality, Quantity, and Cost of Physicians' Prescribing: A Systematic Review
Source: PLoS Med. 2010 Oct 19;7(10):e1000352. doi: 10.1371/journal.pmed.1000352 (PMC2957394; doi:10.1371/journal.pmed.1000352)
Supplement: Alternative Language Abstract S2 — French translation of the abstract by AIV. (0.05 MB DOC) [file pmed.1000352.s002.doc]

**Information fournie par les laboratoires pharmaceutiques et qualité, quantité et coût des prescriptions des médecins: une synthèse de la littérature**

**Résumé**

**Introduction**

Les laboratoires pharmaceutiques ont d**é**pensé 57,5 milliards de dollars pour la promotion pharmaceutique aux États-Unis en 2004. L'industrie assure que les activités promotionnelles fournissent des informations scientifiques et éducatives aux médecins. Bien que certains éléments de preuve indiquent que la promotion peut influer négativement la prescription, les médecins ont des opinions partagées sur la promotion pharmaceutique. L'objectif de cette synthèse de la littérature est d' examiner la relation entre l'exposition à l'information des laboratoires pharmaceutiques et la qualité, la quantité et le coût des prescriptions des médecins.

Méthodes et Résultats

Nous avons recherché les études incluant des médecins prescripteurs exposés à l'information fournie par les laboratoires pharmaceutiques (promotionnelle ou autre). L'exposition comprenait les visites par les représentants pharmaceutiques, les annonces publicitaires, la participation à des réunions parrainées par l’industrie pharmaceutique, l’envoi de prospectus promotionnels, les logiciels de prescription et la participation a des essais cliniques parrainés. Les résultats ont été mesurés en termes de qualité, quantité et coût de prescription des médecins.

Nous avons cherché dans Medline (1966 - Février 2008), International Pharmaceutical Abstracts (1970-Février 2008), EMBASE (1997-Février 2008), Current Contents (de 2001 à 2008) et Central (The Cochrane Library Issue 3, 2007) en utilisant les termes développés avec une bibliothécaire spécialiste. En outre, nous avons examiné les listes de référence et contacté les experts et les laboratoires pharmaceutiques pour obtenir des renseignements.

Les études comparatives randomisées et les études observationnelles évaluant l'information fournie par les laboratoires pharmaceutiques et des mesures de prescription des médecins ont été examinées indépendamment pour leur qualité méthodologique par deux auteurs. Les études étaient exclues si l’information présentée était insuffisante pour évaluer leur qualité. Le texte intégral de 255 articles a été extrait des bases de données électroniques (7185 etudes) et d'autres sources (138 études). Les articles ont ensuite été exclus si ils ne remplissaient pas les critères d'inclusion (179) ou les critères d'évaluation de la qualité (18), laissant un total de 58 études avec 87 analyses distinctes. Les données ont été extraites indépendamment par deux auteurs et une synthèse narrative effectuée suivant les recommandations Moose.

L'exposition à l'information des laboratoires pharmaceutiques a été associée à une baisse de la qualité de la prescription ou aucune association n’a été observée à une exception près, à une fréquence plus élevée de la prescription ou aucune association, et à une hausse des coûts de prescription ou aucune association à une exception près. La synthèse narrative des résultats de la recherche a été complementée par une méta-analyse des études sur la fréquence des prescriptions qui a trouvé une forte hétérogénéité entre les études.

Le caractère observationnel de la plupart des études incluses est la principale limitation de cette synthèse.

**Conclusions**

A de rares exceptions près, les études d'exposition à l’information fournie directement par les laboratoires pharmaceutiques ont montré des associations avec une fréquence de prescription plus élevée, des coûts de prescription plus élevés, ou une qualité de prescription inférieure ou n’ont pas montré d’association. Nous n'avons pas trouvé de preuve d'une nette amélioration de la prescription, mais la littérature disponible ne permet pas d’exclure la possibilité que la prescription puisse parfois être améliorée. Nous recommandons aux praticiens de suivre le principe de précaution et donc d’éviter l'exposition à l'information provenant des laboratoires pharmaceutiques.
