# Supplementary material for: Information from Pharmaceutical Companies and the Quality, Quantity, and Cost of Physicians' Prescribing: A Systematic Review
Source: PLoS Med. 2010 Oct 19;7(10):e1000352. doi: 10.1371/journal.pmed.1000352 (PMC2957394; doi:10.1371/journal.pmed.1000352)
Supplement: Alternative Language Abstract S3 — Spanish translation of the abstract by Diana L. Matallana. (0.05 MB DOC) [file pmed.1000352.s003.doc]

|  |  |
| --- | --- |
| Abstract | Resumen |
|  |  |
| Background | Antecedentes |
|  |  |
| Pharmaceutical companies spent $57.5 billion on pharmaceutical promotion in the United States in 2004. The Industry claims that promotion provides scientific and educational information to physicians. While some evidence indicates that promotion may adversely influence prescribing, physicians hold a wide range of views about pharmaceutical promotion. The objective of this review is to examine the relationship between exposure to information from pharmaceutical companies and the quality, quantity and cost of physicians’ prescribing. | Las compañías farmacéuticas gastaron en promoción $57,5 mil millones dólares en los Estados Unidos en 2004. La industria discute que la promoción ofrece información científica y educativa a los médicos. Mientras que algunas evidencias indican que esta promoción puede influir, adversamente la prescripción, los médicos tienen una amplia gama de puntos de vista sobre la promoción farmacéutica. El objetivo de esta revisión es el de examinar la relación entre la exposición de los médicos a la información de las compañías farmacéuticas, así como la calidad, cantidad y costo de la prescripción que realizan los médicos. |
|  |  |
| Methods and Findings | Métodos y Resultados |
| We searched for studies of physicians with prescribing rights who were exposed to information from pharmaceutical companies (promotional or otherwise). Exposures included pharmaceutical sales representative visits, journal advertisements, attendance at pharmaceutical sponsored meetings, mailed information, prescribing software and participation in sponsored clinical trials. The outcomes measured were quality, quantity and cost of physicians’ prescribing. | Se buscaron estudios con derechos de prescripción, de galenos que fueron expuestos a la información de las compañías farmacéuticas (de promoción o de otro tipo). Las exposiciones incluyeron visitas farmacéuticas de representantes de ventas, anuncios en revistas científicas, la asistencia a reuniones patrocinadas por la industria farmacéutica, información enviada por correo, prescripción por software y la participación en ensayos clínicos patrocinados. Los resultados medidos fueron la calidad, cantidad y costo de las prescripciones de los médicos. |
|  |  |
| We searched Medline (1966- February 2008), International Pharmaceutical Abstracts (1970-February 2008), Embase (1997-February 2008), Current Contents (2001 to 2008) and Central (The Cochrane Library Issue 3, 2007) using the search terms developed with an expert librarian. Additionally, we reviewed reference lists and contacted experts and pharmaceutical companies for information. | Se hicieron búsquedas en Medline (1966 - febrero de 2008), International Pharmaceutical Abstracts (1970-febrero 2008), Embase (1997-febrero 2008), Current Contents (2001 a 2008) y Central (The Cochrane Library, Número 3, 2007) utilizando términos de búsqueda desarrollado con un bibliotecario experto. Adicionalmente, se revisaron las listas de referencia y contacto con expertos y compañías farmacéuticas para obtener información. |
|  |  |
| Randomized controlled and observational studies evaluating information from pharmaceutical companies and measures of physicians’ prescribing were independently appraised for methodological quality by two authors. Studies were excluded where insufficient study information precluded appraisal. The full text of 255 articles was retrieved from electronic databases (7185 studies) and other sources (138 studies). Articles were then excluded because they did not fulfil inclusion criteria (179) or quality appraisal criteria (18) leaving 58 included studies with 87 distinct analyses. Data were extracted independently by two authors and a narrative synthesis performed following the MOOSE guidelines. | Estudios controlados aleatorios y de observación evaluaron la información de las compañías farmacéuticas y las medidas de las prescripciones médicas de prescripción se evaluaron de forma independiente para garantizar la calidad metodológica de dos autores. Se excluyeron los estudios donde había insuficiente información. El texto completo de 255 artículos fue recuperado de bases de datos electrónicas (7185 estudios) y de otras fuentes (138 estudios). Los artículos fueron excluidos luego cuando no se cumplían los criterios de inclusión (179) o criterios de calidad de evaluación (18), dejando 58 estudios que incluyeron 87 análisis diferentes. Los datos fueron extraídos de forma independiente por dos autores y una síntesis narrativa se llevó a cabo siguiendo las directrices de MOOSE. |
|  |  |
| Exposure to information from pharmaceutical companies was associated with lower quality prescribing or no association with one exception, higher frequency of prescribing or no association and higher prescribing costs or no association with one exception. The narrative synthesis finding of variable results was supported by a meta-analysis of studies of prescribing frequency that found significant heterogeneity. | La exposición a la información de las compañías farmacéuticas se asoció con una menor calidad de prescripción o con ninguna asociación con una excepción, una mayor frecuencia de prescripción o con ninguna asociación y mayores costos de prescripción o con ninguna asociación con una excepción. Los hallazgos de la síntesis narrativa de los resultados variables se realizaron a través de un meta-análisis de estudios de la frecuencia de prescripción y se encontró una heterogeneidad significativa. |
|  |  |
| The observational nature of most included studies is the main limitation of this review. | La naturaleza de observación de la mayoría de los estudios incluidos es la principal limitación de esta revisión. |
|  |  |
| Conclusions | Conclusiones |
|  |  |
| With rare exceptions studies of exposure to information provided directly by pharmaceutical companies have found associations with higher prescribing frequency or higher costs or lower prescribing quality or have not found significant associations. We did not find evidence of net improvements in prescribing but the available literature does not exclude the possibility that prescribing may sometimes be improved. We recommend that practitioners follow the precautionary principle and thus avoid exposure to information from pharmaceutical companies. | Con raras excepciones, en los estudios que incluyeron exposición a la información proporcionada directamente por las compañías farmacéuticas se encontraron asociaciones con mayor frecuencia a la prescripción de la información, mayores costos o menor calidad de la prescripción o no se encontraron asociaciones significativas. No hemos encontrado pruebas de una mejoría neta en la prescripción, pero la literatura disponible no excluye la posibilidad de que la prescripción a veces se puede mejorar. Se recomienda que los médicos siguan el principio de precaución y así evitar la exposición a la información de las compañías farmacéuticas. |

Abstract

Background

Pharmaceutical companies spent $57.5 billion on pharmaceutical promotion in the United States in 2004. The Industry claims that promotion provides scientific and educational information to physicians. While some evidence indicates that promotion may adversely influence prescribing, physicians hold a wide range of views about pharmaceutical promotion. The objective of this review is to examine the relationship between exposure to information from pharmaceutical companies and the quality, quantity and cost of physicians’ prescribing.

Methods and Findings

We searched for studies of physicians with prescribing rights who were exposed to information from pharmaceutical companies (promotional or otherwise). Exposures included pharmaceutical sales representative visits, journal advertisements, attendance at pharmaceutical sponsored meetings, mailed information, prescribing software and participation in sponsored clinical trials. The outcomes measured were quality, quantity and cost of physicians’ prescribing.

We searched Medline (1966- February 2008), International Pharmaceutical Abstracts (1970-February 2008), Embase (1997-February 2008), Current Contents (2001 to 2008) and Central (The Cochrane Library Issue 3, 2007) using the search terms developed with an expert librarian. Additionally, we reviewed reference lists and contacted experts and pharmaceutical companies for information.

Randomized controlled and observational studies evaluating information from pharmaceutical companies and measures of physicians’ prescribing were independently appraised for methodological quality by two authors. Studies were excluded where insufficient study information precluded appraisal. The full text of 255 articles was retrieved from electronic databases (7185 studies) and other sources (138 studies). Articles were then excluded because they did not fulfil inclusion criteria (179) or quality appraisal criteria (18) leaving 58 included studies with 87 distinct analyses. Data were extracted independently by two authors and a narrative synthesis performed following the MOOSE guidelines.

Exposure to information from pharmaceutical companies was associated with lower quality prescribing or no association with one exception, higher frequency of prescribing or no association and higher prescribing costs or no association with one exception. The narrative synthesis finding of variable results was supported by a meta-analysis of studies of prescribing frequency that found significant heterogeneity.

The observational nature of most included studies is the main limitation of this review.

Conclusions

With rare exceptions studies of exposure to information provided directly by pharmaceutical companies have found associations with higher prescribing frequency or higher costs or lower prescribing quality or have not found significant associations. We did not find evidence of net improvements in prescribing but the available literature does not exclude the possibility that prescribing may sometimes be improved. We recommend that practitioners follow the precautionary principle and thus avoid exposure to information from pharmaceutical companies.
