# Supplementary material for: Information from Pharmaceutical Companies and the Quality, Quantity, and Cost of Physicians' Prescribing: A Systematic Review
Source: PLoS Med. 2010 Oct 19;7(10):e1000352. doi: 10.1371/journal.pmed.1000352 (PMC2957394; doi:10.1371/journal.pmed.1000352)
Supplement: Text S1 — MOOSE checklist. (1.72 MB PDF) [file pmed.1000352.s004.pdf]

MOOSE Checklist

**Reporting of background should include**	
Problem definition	√
Hypothesis statement	√
Description of study outcome(s)	√
Type of exposure or intervention used	√
Type of study designs used	√
Study population	√
**Reporting of search strategy should include**	
Qualifications of searchers (eg, librarians and investigators)	√
Search strategy, including time period included in the synthesis and keywords	√
Effort to include all available studies, including contact with authors	√
Databases and registries searched	√
Search software used, name and version, including special features used (eg, explosion)	√
Use of hand searching (eg, reference lists of obtained articles)	√
List of citations located and those excluded, including justification	√
Method of addressing articles published in languages other than English	√
Method of handling abstracts and unpublished studies	√
Description of any contact with authors	√
**Reporting of methods should include**	
Description of relevance or appropriateness of studies assembled for assessing the hypothesis to be tested	√
Rationale for the selection and coding of data (eg, sound clinical principles or convenience)	√
Documentation of how data were classified and coded (eg, multiple raters, blinding, and interrater reliability)	√
Assessment of confounding (eg, comparability of cases and controls in studies where appropriate)	√
Assessment of study quality, including blinding of quality assessors; stratification or regression on possible predictors of study results	√
Assessment of heterogeneity	√
Description of statistical methods (eg, complete description of fixed or random effects models, justification of whether the chosen models account for predictors of study results, dose-response models, or cumulative meta-analysis) in sufficient detail to be replicated	√
Provision of appropriate tables and graphics	√
**Reporting of results should include**	
Graphic summarizing individual study estimates and overall estimate	√
Table giving descriptive information for each study included	√
Results of sensitivity testing (eg, subgroup analysis)	√
Indication of statistical uncertainty of findings	√
**Reporting of discussion should include**	
Quantitative assessment of bias (eg, publication bias)	√
Justification for exclusion (eg, exclusion of non–English-language citations)	√
Assessment of quality of included studies	√
**Reporting of conclusions should include**	
Consideration of alternative explanations for observed results	√
Generalization of the conclusions (ie, appropriate for the data presented and within the domain of the literature review)	√
Guidelines for future research	x
Disclosure of funding source	√
